# Short-, medium-, versus long-chain fatty acids: mechanisms of immunomodulation and disease pathogenesis

**DOI:** 10.1038/s41423-026-01412-z

**Published:** 2026-04-30

**Authors:** Chang H. Kim

**Affiliations:** 1 Department of Pathology, University of Michigan School of Medicine, Ann Arbor, MI 48109 USA; 2 Mary H. Weiser Food Allergy Center, Ann Arbor, MI 48109 USA

**Keywords:** Short-chain fatty acids, Medium-chain fatty acids, Saturated fatty acids, Polyunsaturated fatty acids, Immune regulation, Mucosal immunology, Inflammation

## Abstract

Fatty acids, such as short-chain fatty acids, medium-chain fatty acids and long-chain fatty acids, exist in different chain lengths and with various modifications, which determine their physical, metabolic and biological properties. They serve as important nutrients in energy production via mitochondrial beta-oxidation in various cell types including immune cells. At optimal levels in the body, fatty acids support normal differentiation and function of immune cells. However, at excessive levels, they can cause dysregulation of immune cells and inflammation. The three types of fatty acids regulate cells, in part, via the activation of G protein-coupled receptors, such as GPR41, GPR43, GPR109A, and Olfr78 for short-chain, GPR40 and GPR120 for both medium- and long-chain fatty acids, and GPR84 for medium-chain fatty acids. Activation of these receptors by fatty acids regulates cell proliferation and cell-specific functions. Importantly, fatty acids induce the production of glucagon-like peptide-1 and glucose-dependent insulinotropic polypeptide through activation of G-protein coupled receptors. Short-chain fatty acids additionally control epigenetic regulators such as histone deacetylases and histone acetyltransferases. Saturated long-chain fatty acids and omega-6 polyunsaturated fatty acids are implicated in metabolic diseases and inflammatory conditions, whereas short-chain fatty acids, monounsaturated fatty acids, and omega-3 polyunsaturated fatty acids are generally associated with functional immunity with anti-inflammatory effects. This article explores how fatty acids regulate the immune system, focusing on their common and unique roles, as well as their opposing functions.

## Introduction

Major fatty acids include short-chain fatty acids (SCFAs), medium-chain fatty acids (MCFAs) and long-chain fatty acids (LCFAs), which share a common structure of a hydrocarbon backbone with various lengths and a carboxylic acid group (-COOH) at one end. SCFAs, with 2-6 carbon length, are primarily derived from the intestinal lumen following bacterial fermentation of complex carbohydrates. They include acetic acid (C2), propionic acid (C3), and butyric acid (C4). SCFAs not only support the maturation and activation of the immune system but also play active roles in the suppression of inflammatory responses [1, 2].

MCFAs, with 7–12 carbon-length, are absorbed from certain plant-based foods and animal-derived products. They include caprylic acid (C8), capric acid (C10), and lauric acid (C12). MCFAs, particularly short MCFAs such as C7-C10, are more soluble than LCFAs and are absorbed into the portal vein without the need for chylomicron formation. However, the longer lauric acid (C12) behaves more like a LCFA in its absorption. MCFAs are rapidly processed in the liver, and their mitochondrial oxidation is independent of carnitine [3]. MCFAs can be used by immune cells as an energy source and have mixed immunoregulatory effects, including both anti- and pro-inflammatory actions depending on context, dose, and cell type [4].

Saturated LCFAs (SFAs) with 13-21 carbon-length, such as palmitic acid (16 C:0), stearic acid (18 C:0), and arachidic acid (20 C:0), are primarily obtained from animal fats and certain plant-derived oils in diet [5]. Palmitic acid is also made in the body to store energy from excess glucose. Monounsaturated LCFAs (MUFAs) include the ω-9 oleic acid (C18:1), ω-7 palmitoleic acid (C16:1) and others. Additionally, omega-3 polyunsaturated fatty acids (PUFAs) include eicosapentaenoic acid (EPA), docosahexaenoic acid (DHA), and α-linolenic acid, and omega-6 PUFAs include linoleic acid and arachidonic acid. LCFAs are the basic units of larger lipid molecules, such as triglycerides, phospholipids, and cholesteryl esters. LCFAs are important for energy storage, cell membrane structure and serve as precursors for signaling molecules [6]. Compared to SCFAs and MCFAs, LCFAs are less soluble, travel through the lymphatic system in chylomicrons, and require the carnitine shuttle to enter the mitochondria for oxidation [7]. LCFAs regulate the immune system by acting as either anti-inflammatory or pro-inflammatory signaling molecules depending on subtype [8].

Decades of research have revealed important functions of fatty acids in both metabolic and immune regulation [9]. They provide energy and regulate metabolic processes in cells. They trigger activation of various GPCRs to regulate cells, induce metabolic hormones, and modulate immune responses through receptor signaling. They can exert distinct functions in part through their specificity in receptor usage and metabolic as well as physical properties. Moreover, they are precursors of many metabolites with additional receptors and functions. The major focus of this review is to contrast the differences among these fatty acids in terms of their regulatory functions in the immune system and discuss known action mechanisms.

## Sources, transport, and utilization of short-, medium- and long-chain fatty acids

**Fig. 1:**
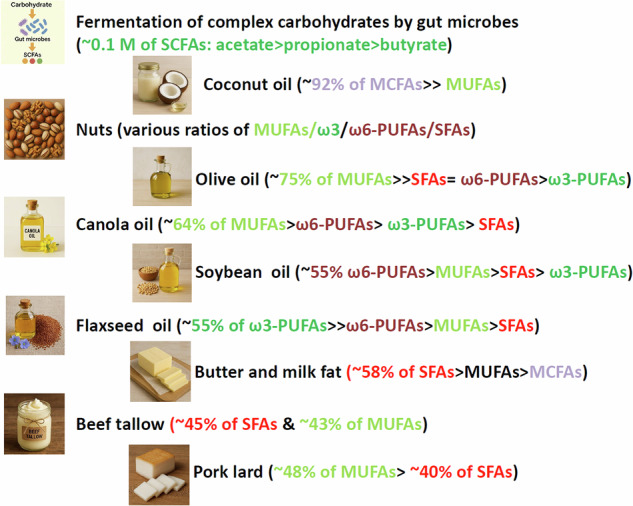
Common sources of major types of fatty acids. Short-chain fatty acids are derived from carbohydrates that reach the colon for fermentation. Usually, digestion-resistant and complex carbohydrates such as dietary fibers are the prebiotics that are utilized by gut microbes to produce SCFAs. Acetate, propionate and butyrate are the most common SCFAs in the colon. MCFAs are abundant in certain plant-based oils, eggs, milk, and butter. LCFAs are divided into saturated fatty acids (SFAs), monounsaturated fatty acids (MUFAs) and polyunsaturated fatty acids (PUFAs). The SFA palmitic acid is also produced de novo from glucose in cells. PUFAs are further divided into multiple fatty acids (e.g., omega-3 and omega-6) depending on the location and number of double bonds in the hydrocarbon chain. Many plant-based oils contain omega-3 and omega-6 fatty acids at various ratios. Animal fats are mostly composed of SFAs and MUFAs. Coconut oils and dairy fat products contain relatively high levels of MCFAs. Therefore, diets primarily determine the amount and composition of different types of fatty acids available for absorption in the intestine

The three types of fatty acids, despite their shared structural features, are different in their sources, absorption into the body, and metabolism (Fig. 1). SCFAs are derived mainly from microbial fermentation in the gut, whereas medium-chain and long-chain fatty acids are largely derived from food. Because of the differences in their size and solubility in the aqueous phase, they are taken up and metabolized in somewhat different manners. First, SCFAs are mainly produced from carbohydrates that reach the colon [1]. Hard to digest dietary fibers and resistant starches are the major prebiotics that feed gut microbes to recognize, degrade and metabolize to produce SCFAs. While 1-6 carbon length fatty acids are called SCFAs, three SCFAs (acetate, propionate and butyrate) are dominant in the mammalian colon, reaching more than 0.1 M combined concentrations [10]. These three SCFAs are considered functionally important SCFAs.

SCFAs are produced by several different pathways in bacteria [11–13]. Certain acetogenic bacteria, such as those in the *Clostridium* and *Blautia* genera, can produce acetate from hydrogen and carbon dioxide or from formic acid via the Wood-Ljungdahl Pathway or fermentation through acetyl-CoA. Propionate is produced by the succinate (*Bacteroidetes* phylum), acrylate (*Veillonellaceae* and *Lachnospiraceae* families) and propanediol (some *Proteobacteria* and *Lachnospiraceae* species) pathways. Butyrate is made via the Butyryl-CoA:Acetate CoA-Transferase Pathway (*Faecalibacterium*, *Eubacterium*, and *Roseburia)* and the Butyrate Kinase Pathway (some *Coprococcus* species). The SCFAs produced in the colon are absorbed and used by microbes and colonocytes. Some of the SCFAs absorbed into colonocytes are transported out of the cells into blood vessels (e.g., portal vein) to reach the liver and the whole body [1, 11–13]. SCFAs are expected to have the most pronounced effects in the colon, but it has been extensively reported that they exert their effects across the whole body by supporting barrier tissue functions and regulating diverse cell types, including immune cells [2, 14–16]. They also regulate the central nervous system and metabolism and serve as building blocks to make glucose, cholesterol, and fats.

**Fig. 2:**
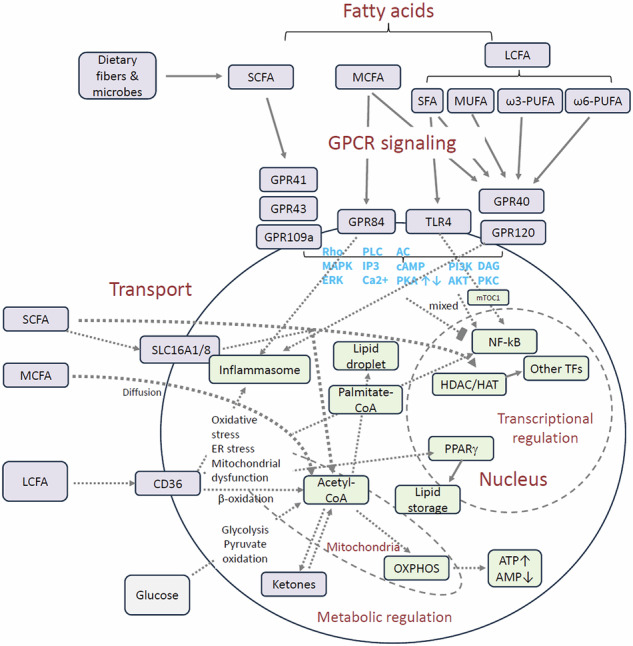
Transport and cellular targets of major fatty acids. Fatty acids affect various cell types, including immune cells through several different ways. One way is to integrate into cell membranes and affect membrane protein functions, such as TLR4 activation by SFAs. Fatty acid transport into the cell cytoplasm involves diffusion and/or protein transporters, such as CD36 for LCFAs. CD36 activation is also involved in cellular lipotoxicity, triggering oxidative stress, ER stress, mitochondrial dysfunction, cell death, and inflammation (inflammasome and NF-κB activation). Fatty acids activate GPCRs (GPR41, GPR43, and GPR109A by SCFAs; GPR84 by MCFAs, GPR40 and GPR120 by LCFAs and MCFAs) on the cell membrane for intracellular signaling in a cell type and fatty acid-dependent manner. Once inside cells, fatty acids are converted into acetyl-CoA, which fuels oxidative phosphorylation in mitochondria to produce ATP and decrease AMP levels. Acetyl-CoA can be converted to ketones such as β-hydroxybutyrate when glucose levels are limited due to low carbohydrate consumption. β-hydroxybutyrate has similar effects as the SCFA butyrate in that it suppresses HDACs and triggers GPCRs. HDAC inhibition and HAT activation by SCFAs support cell activation and differentiation in diverse cells. Related omega-3 PUFAs (e.g., DHA and EPA) are metabolized into biologically active derivatives (e.g., resolvins and protectins) that can inhibit inflammatory transcription factors (e.g., NF-κB and AP-1) and bind to receptors, such as PPAR-γ and GPR120 to further affect immune cell activities. Omega-6 PUFAs can be metabolized to eicosanoids, such as prostaglandins, thromboxanes (2-series), leukotrienes (4-series), hydroxyeicosatetraenoic acids, epoxyeicosatrienoic acids, and lipoxins, which subsequently regulate immune cells via additional receptors. Acetyl-CoA is also made from glucose and used for de novo synthesis of palmitic acid in cells. NF-κB activation by SFAs promotes inflammation. SREBP1a activation by SFAs promotes triglyceride synthesis and lipid storage. Abbreviations: AC (adenylyl cyclase), DAG (diacylglycerol), ER (endoplasmic reticulum), IP3 (inositol 1,4,5-trisphosphate), PLC (phospholipase C), and ROS (reactive oxygen species)

SCFAs are used as energy sources for many cell types, including colonocytes and immune cells [17, 18]. They can be converted into acetyl-CoA (Fig. 2), which enters the citric acid cycle for the generation of ATP or can be used to synthesize LCFAs [19]. Propionate is converted to succinyl-CoA, which enters the citric acid cycle to eventually produce oxaloacetate, which is a key to the production of glucose [20, 21]. SCFAs inhibit histone deacetylases (HDACs) and activate acetyltransferases (HATs) [22, 23]. SCFAs inhibit NF-κB but promote HIF-1α activity [24, 25]. Moreover, SCFAs bidirectionally regulate NLRP3 inflammasome in a context dependent manner [26, 27]. These interactions trigger epigenetic regulation and changes in immune and metabolic functions.

MCFAs are primarily found as components of medium-chain triglycerides (MCTs) in foods, such as coconut oil, palm kernel oil, and dairy fats and are also obtained from breast milk in babies. MCTs are digested to free up MCFAs from glycerol. Lingual and gastric lipases begin to break down MCTs in the stomach [28]. In the duodenum, pancreatic lipases finish the process, rapidly hydrolyzing MCTs into free MCFAs. The major MCFAs in the intestinal lumen are caprylic acid (C8:0), capric acid (C10:0), and lauric acid (C12:0) [29]. Unlike LCFAs, MCFAs do not require bile salts for digestion. MCFAs are absorbed by intestinal cells via passive diffusion as well as active transport mediated by the fatty acid transport protein 4 (FATP4) [30, 31]. Once inside the intestinal cells, MCFAs are transported via the portal vein to the liver for metabolism. Once in the liver, MCFAs are quickly oxidized for energy, or converted into ketone bodies, rather than being stored as fatty acyl-CoA.

LCFAs are derived mostly from long-chain triglycerides (LCTs) consumed from a diet rich in fats and oils, particularly from animal and plant sources such as meat, vegetable oils (e.g., olive oil and soybean oil), eggs, fish, milk, nuts, and seeds (Fig. 1). Some unsaturated LCFAs, such as omega-3 and omega-6 fatty acids, are not readily produced in the human body and must be obtained through food [32]. Dietary fats are broken down in the small intestine, then get absorbed by enterocytes, reassembled, and packaged into chylomicrons for transport into the lymphatic and circulatory systems. The process begins with emulsification by bile salts and digestion by lipases, creating micelles that deliver LCFAs to the intestinal wall. Once inside cells, LCFAs are re-esterified into triglycerides and packaged into chylomicrons before being released into the lymph and eventually into the blood. Free fatty acid (FFA) concentration in blood reaches 0.1 to 0.6 mM and that of LCTs is less than 1.7 mM in healthy people [33]. FFAs are released from stored triglycerides in adipose tissues through a process called lipolysis. The released FFAs enter the bloodstream, where they bind to albumin for transport to various tissues for utilization or storage [34].

Cells take up SCFAs and MCFAs via passive diffusion and specific transport proteins. MCT1 and SMCT1 transport SCFAs into cells [35–37]. LCTs are packaged into large lipoprotein particles or chylomicron, which are drained from the intestine through lymphatic vessels to the blood circulation. The uptake of LCFAs into the enterocyte involves proteins like FATP4 and CD36, which are involved in LCFA transport into other cells such as adipocytes, muscle cells, macrophages and endothelial cells [30, 38]. Once LCFAs cross the plasma membrane, they are immediately bound by cytoplasmic fatty acid-binding proteins (FABPs), which act as carriers to shuttle the LCFAs to their destinations within the cell, such as for metabolism (oxidation or esterification) or for synthesis into other cellular components. Inside the cell, FFA is conjugated to Coenzyme A for beta-oxidation in mitochondria and peroxisomes [39]. Beta-oxidation directly yields acetyl-CoA, nicotinamide adenine dinucleotide (NADH), flavin adenine dinucleotide (FADH2), and ATP. Acetyl-CoA enters the citric acid cycle to generate ATP through oxidative phosphorylation (Fig. 2). Acetyl-CoA is also used for de novo lipogenesis of palmitic acid in cells for storage [40].

The different fatty acids have considerable implications in cardiovascular, metabolic, and general human health [13, 41]. Saturated LCFAs tend to raise low-density lipoprotein (LDL) cholesterol and can increase cardiovascular risk. MUFAs are generally associated with improved lipid profiles and cardiometabolic health, often lowering LDL while maintaining or improving high-density lipoprotein (HDL) levels and supporting better insulin sensitivity. PUFAs, such as omega-6 (e.g., soybean, corn, and sunflower oils) and omega-3 fats (e.g., fish, flax, and walnut oils) lower LDL and reduce heart disease risk, while omega-3s additionally support triglyceride lowering and have anti-inflammatory and vascular benefits [41]. Short-chain fatty acids (SCFAs), primarily acetate, propionate, and butyrate, have diverse health benefits, including improving gut health, exerting anti-inflammatory effects, modulating appetite signaling and glucose metabolism [13].

## Fatty acid-sensing cell surface GPCRs

What makes fatty acids more than nutritional substances or building blocks is their function in activating GPCRs (Fig. 2). SCFAs serve as ligands for several GPCRs such as GPR41, GPR43, GPR109A, and Olfr78. These receptors are expressed by distinct cell types: GPR41 (adipocytes, intestinal enteroendocrine cells, epithelial cells, pancreas cells, neuronal cells, cardiomyocytes, and vascular cells), GPR43 (ILC3, ILC2, neutrophils, adipocytes, intestinal enteroendocrine cells, epithelial cells, and pancreas cells), GPR109A (neutrophils, macrophages, adipocytes, and microglial cells) and Olfr78 (kidney cells, vascular smooth muscle cells, and intestinal enteroendocrine cells) [1, 15, 42–49]. Depending on the cell types that express these receptors, the activation of these receptors results in unique biological consequences (Fig. 2).

Compared with SCFAs, MCFAs and LCFAs use different receptors [50]. They trigger GPR40 and GPR120. Additionally, MCFAs such as decanoic acid (C10) and undecanoic acid (C11) along with their hydroxylated forms, but not LCFAs, trigger GPR84 [51]. Also, bacterial quorum-sensing molecules such as cis or trans-2-decenoic acid (cis-2-C10) serve as additional ligands for GPR84 [52]. GPR119 is a receptor mainly for LCFA derivatives, such as oleoylethanolamide (OEA) and lysophosphatidylcholine (LPC), and related lipid signaling molecules [53–55]. These receptors are expressed by pancreatic beta cells (GPR119), enteroendocrine cells (GPR119, GPR120), intestinal epithelial cells (GPR84), adipocytes (GPR84), macrophages (GPR84, GPR120), T cells (GPR84), neutrophils (GPR84), dendritic cells (GPR84, GPR120), and B cells (GPR40) [50–52, 56, 57]. They are also expressed in microglial cells (GPR84, GPR120) and neuronal cells (GPR119) potentially controlling inflammation, pain sensing and memory [58].

The FA-binding GPCRs signal through G proteins such as Gα_i_, Gα_o_, Gα_s_, and Gα_q_ [59]. Activation of these G proteins leads to the dissociation of Gα and Gβγ subunits. Gα_s_ stimulates adenylyl cyclase, increasing cAMP and protein kinase A (PKA) activity. In contrast, Gα_i_ and Gα_o_ inhibit adenylyl cyclase, lowering cAMP levels [60]. Gα_q_ activates phospholipase C (PLC), raising intracellular Ca²⁺ and activating protein kinase C (PKC). In some cases, the Gβγ subunit can activate PLC or PI3K, depending on cell type and receptor context. GPR41 and GPR43 signal through the Gi/o family of G proteins [61]. GPR43 additionally activates Gq. GPR109A acts via Gα_i_, reducing intracellular cAMP. GPR84 mainly couples to Gi/o, while GPR40 couples to both Gα_q_ and Gα_s_ [62–64]. GPR119 activates Gα_s_, increasing cyclic AMP [65]. GPR120 primarily signals via β-arrestin-2 and Gα_q_, the latter of which activates PLC and increases intracellular calcium [66].

## Multi-level and differential regulation of immune cells and inflammation by SCFAs, MCFAs, and LCFAs

**Fig. 3:**
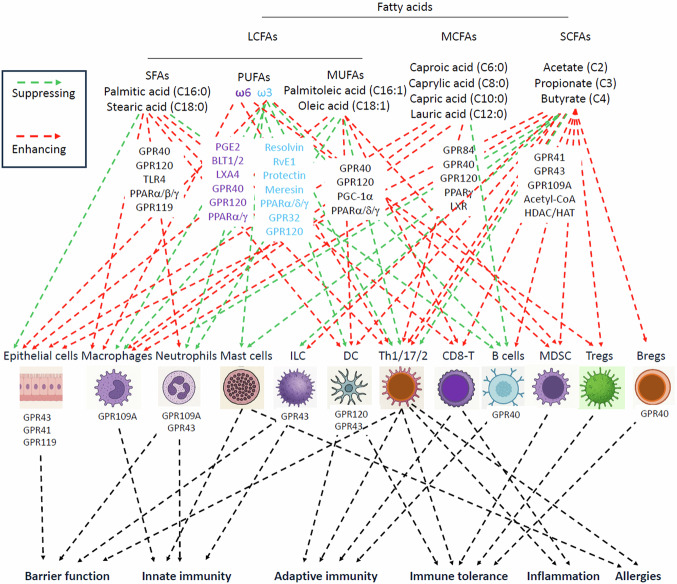
Immune regulation by fatty acids and their metabolites. Fatty acids have significant effects on immune cells. SCFAs, MCFAs, and LCFAs activate shared and distinct surface receptors for activating various cell types, including immune cells. LCFAs and MCFAs are integrated into the cell membrane to influence cell activation and signaling status. They are also metabolized to produce many different metabolites which activate additional receptors that are expressed in a relatively specific manner depending on cell type. As a result, the three types of LCFAs, SFAs, omega-6 PUFAs, and omega-3 PUFAs exert different effects on the immune system, with the former two having largely inflammatory effects, while omega-3 PUFAs having overall anti-inflammatory effects. Unlike LCFAs, which are readily stored in adipocytes and other cells, MCFAs are readily metabolized in cells. This property of MCFAs is important for their beneficial effects on metabolism and immune system, more likely suppressing obesity and inflammatory diseases. Likewise, SCFAs activate GPCRs on cells or are rapidly absorbed into cells for intracellular regulation of metabolism and gene expression. They play important roles in supporting barrier epithelial cells, promoting effector immune cells and inducing regulatory immune cells to prevent inflammatory diseases

Most fatty acids have the potential to regulate immune cells through multiple mechanisms, including receptor-mediated signaling, metabolic regulation, and epigenetic modifications (Fig. 3). They also affect immune cells through indirect regulation of barrier function, metabolic hormone secretion, and adipocytes. In addition, saturated (e.g., palmitic and stearic acids) and unsaturated LCFAs, including omega-3 fatty acids (e.g, DHA/EPA) and omega-6 fatty acids (e.g., arachidonic acid), can be integrated into cell membranes, thereby differentially regulating membrane fluidity and the function of various membrane-bound proteins and receptors [67, 68]. LCFAs can exert different effects on cells depending on whether they are saturated or unsaturated and location and number of double bonds in the carboxylic chain (omega-3 vs. omega-6). SFAs promote the activation of the NOD-, LRR- and pyrin domain-containing protein 3 (NLRP3) inflammasome in macrophages through indirect mechanisms such as altering membrane ion efflux, activating NF-κB, and causing endoplasmic reticulum (ER) stress and mitochondrial dysfunction [69–71]. Importantly, biologically active metabolites such as anti-inflammatory EPA and DHA are produced from omega-3, whereas pro-inflammatory arachidonic acid (n-6) is produced from omega-6 LCFAs, primarily linoleic acid (Table 1) [72].

**Table 1:** Major fatty acids and their metabolites with immunological effects

	Fatty acids or their metabolites	Targets	Key Immune Actions
SCFAs	Acetate (C2) Propionate (C3) Butyrate (C4)	GPR43, GPR41, GPR109A (butyrate), Olfr78 (propionate), HDACs, HATs	Supports barrier cells and immune cells by providing Acetyl-CoA for the TCA cycle and GPCR signaling; Increase gene expression to control cell metabolism and differentiation; Promote both effector T cells and regulatory T cells depending on the context.
MCFAs	Lauric acid (C12)	PPARα/γ TLR4, NF-κB Mitochondria	Antimicrobial by membrane disruption; Anti-inflammatory; Increasing oxidative phosphorylation; Promotes effector T cells
	Capric acid (C10)	Mitochondria, PPARα	Promote fatty acid oxidation; Decreases ROS; Antimicrobial effects; Anti-inflammatory
	Caprylic acid (C8)		
SFAs	Ceramide	PKCζ, PP2A, NF-κB, JNK, Membrane rafts, Caspases	Apoptosis; Cytokine regulation
	Diacylglycerol	PKC, RasGRP, PLC	Immune cell activation and proliferation
	Acylcarnitines	CPT 1/2, PPARs, Mitochondrial stress, Inflammasome	Metabolic inflammation; Immune signaling
	Lysophosphatidylcholine	G2A (GPR132), LPA_1-6_, PLC, PKC, Scramblases	Chemotactic; Inflammatory
	Sphingolipids (e.g., S1P)	S1PR1-5, PI3K/Akt, ERK/MAPK, PKC	Lymphocyte trafficking; Immune modulation
	Endocannabinoids	CB1, CB2, TRPV1, PPARs, MAPK, NF-κB	Immunosuppression; Anti-inflammatory
Omega-3 PUFAs	EPA	PPARs, GPR120, NF-κB, Membrane phospholipids	Anti-inflammatory
	DHA	PPARs, RXR, GPR120, NF-κB, SIRT1	Neuroprotection; Immune modulation
	Resolvins	GPR32, ChemR23, ALX/FPR2, PPARγ	Resolution of inflammation; Pro-resolving
	Protectins	GPR37, PPARγ, NF-κB	Anti-inflammatory; Neuroprotective
	Maresins	LGR6, PPARγ	Pro-resolving,; Tissue repair
	17-HDHA, 18-HEPE	PPARγ, NF-κB	Anti-inflammatory; SPM production
	PGE3	EP GPCRs, NF-κB, MAPK	Immune modulation
	TXA3	Thromboxane receptor (TP)	Immune modulation
	LTB5	BLT1/BLT2, MAPK, Ca²⁺	Chemotactic; Weakly inflammatory or anti-inflammatory
Omega-6 PUFAs	PGE1, PGE2	Prostaglandin E receptors (EP1-EP4)	Th1 suppression; Th17 modulation; Fever and vasodilation
	PGD2	DP1, DP2 (CRTH2)	Eosinophil-Th2 chemotaxis; Allergic inflammation
	TXA2	Thromboxane A2 receptor	Platelet aggregation; Acute inflammation
	LTB4	BLT1, BLT2	Neutrophil chemotaxis and activation
	LTC4/D4/E4	CysLT1, CysLT2	Bronchoconstriction; Inducing allergic responses
	5/11/12/15/20-HETEs	Leukotriene precursors, GPR31, GPR75	Modulate leukocyte activation; Inflammatory; Neutrophil chemotaxis
	Lipoxins	ALX/FPR2	Anti-inflammatory; Resolution of inflammation

The information in this table was retrieved, in part, from the following references: [1] for SCFAs [96–219], for MCFAs [220–222], for SFAs [223, 224], for omega-3 PUFAs [224, 225], for omega-6 PUFAs, and other references described in the main text. Abbreviations: 17-HDHA, 17-hydroxydocosahexaenoic acid; 18-HEPE, 18-hydroxyeicosapentaenoic acid; 5/11/12/15/20-HETEs, 5/11/12/15/20-hydroxyeicosatetraenoic acids; Akt, protein kinase B; ALX/FPR2, lipoxin A4 receptor/formyl peptide receptor 2; BLT1, leukotriene B4 receptor 1; BLT2, leukotriene B4 receptor 2; C10, capric acid; C12, lauric acid; C2, acetate; C3, propionate; C4, butyrate; C8, caprylic acid; Ca²⁺, calcium ion; CB1, cannabinoid receptor 1; CB2, cannabinoid receptor 2; ChemR23, chemerin receptor 23; CPT 1/2, carnitine palmitoyltransferase 1 and 2; CRTH2, chemoattractant receptor-homologous molecule expressed on Th2 cells; CysLT1, cysteinyl leukotriene receptor 1; CysLT2, cysteinyl leukotriene receptor 2; DHA, docosahexaenoic acid; DP1, D prostanoid receptor 1; DP2, D prostanoid receptor 2; EPA, eicosapentaenoic acid; EP GPCRs, E prostanoid G protein-coupled receptors; EP1-EP4, E prostanoid receptors 1 through 4; ERK, extracellular signal-regulated kinase; G2A, G protein-coupled receptor G2A; GPR109A, G protein-coupled receptor 109A; GPR120, G protein-coupled receptor 120; GPR132, G protein-coupled receptor 132; GPR31, G protein-coupled receptor 31; GPR41, G protein-coupled receptor 41; GPR43, G protein-coupled receptor 43; GPR75, G protein-coupled receptor 75; HATs, histone acetyltransferases; HDACs, histone deacetylases; JNK, c-Jun N-terminal kinase; LGR6, leucine-rich repeat-containing G protein-coupled receptor 6; LPA1-6, lysophosphatidic acid receptors 1 through 6; LTB4, leukotriene B4; LTB5, leukotriene B5; LTC4, leukotriene C4; LTD4, leukotriene D4; LTE4, leukotriene E4; MAPK, mitogen-activated protein kinase; NF-κB, nuclear factor kappa-light-chain-enhancer of activated B cells; Olfr78, olfactory receptor 78; PGD2, prostaglandin D2; PGE1, prostaglandin E1; PGE2, prostaglandin E2; PGE3, prostaglandin E3; PI3K, phosphoinositide 3-kinase; PKC, protein kinase C; PKCζ, protein kinase C zeta; PLC, phospholipase C; PPARα, peroxisome proliferator-activated receptor alpha; PPARγ, peroxisome proliferator-activated receptor gamma; PPARs, peroxisome proliferator-activated receptors; PP2A, protein phosphatase 2A; RasGRP, Ras guanyl-releasing protein; RXR, retinoid X receptor; S1P, sphingosine-1-phosphate; S1PR1-5, sphingosine-1-phosphate receptors 1 through 5; SIRT1, sirtuin 1; SPM, specialized pro-resolving mediators; TLR4, Toll-like receptor 4; TP, thromboxane receptor; TRPV1, transient receptor potential vanilloid 1; TXA2, thromboxane A2; TXA3, thromboxane A3

A major functional target of SCFAs is the colon due to their presence in the gut lumen at high levels. SCFAs activate colonocytes via GPR41 and GPR43 [42]. Activation of these receptors is important for preparing colonocytes for optimal barrier function as well as timely epithelial innate immune responses following microbial invasion. Without GPR41 or GPR43, the epithelial immune response to bacterial infection is slow, leading to an inability to clear microbes and chronic inflammatory responses in the colon. The GPR41 and GPR43 signaling allows the mitogen-activated protein kinase (MAPK)-extracellular signal-regulated kinase 1/2 (ERK) pathway to produce acute inflammatory cytokines such as IL-1β, IL-6, and IL-8, which induce neutrophil recruitment and activation to clear invading microbes. Additionally, SCFAs promote the production of antimicrobial factors and regulate the turnover of epithelial cells to strengthen the barrier that prevents microbial invasion. SCFAs modulate the function of innate immune cells such as neutrophils, macrophages, and natural killer cells. SCFAs can directly and indirectly control neutrophil recruitment to inflammatory sites. GPR43 is highly expressed by neutrophils and acts as one of their chemoattractant receptors [45, 73, 74]. Thus, neutrophils may be attracted to high concentrations of SCFAs during acute immune responses [74]. The physiological importance of this function in the steady state, however, is unclear. Contradictorily, GPR43 functions to indirectly suppress chronic neutrophil recruitment to intestinal tissues by strengthening the gut barrier [42, 75].

SCFAs regulate innate lymphoid cells through GPR43 and GPR109A [48, 61]. SCFAs induce proliferation of ILC1, ILC2 and ILC3 in steady states. ILC2 in the lungs under allergic condition can be suppressed by butyrate, and this appears to be mediated by HDAC inhibition [76, 77]. Stimulation of ILC3 by SCFAs can increase IL-22 expression [48, 61], which acts on intestinal epithelial cells to promote the production of anti-microbial peptides.

SCFAs regulate the differentiation, polarization and metabolism of macrophages. They can promote the generation of anti-inflammatory macrophages and enhance their ability to clear pathogens [78, 79] They also suppress pro-inflammatory cytokine responses and inhibit NF-κB signaling and downregulate lipopolysaccharide-induced production of nitric oxide, IL-6, and IL-12 [80]. Butyrate promotes M2 type but inhibits M1 type macrophages induced by lipopolysaccharide (LPS) [81]. SCFAs achieve this by altering gene expression through HDAC inhibition. Additionally, the activation of GPR109A is involved in inducing anti-inflammatory properties in macrophages and dendritic cells for the generation of Treg cells and IL-10-producing T cells [49, 82]. SCFAs are metabolized through oxidative phosphorylation (OXPHOS) and the tricarboxylic acid (TCA) cycle in macrophages to produce energy.

SCFAs support the differentiation of T cells to better equip them for host defense. More specifically, SCFAs promote the differentiation of Th1 and Th17 cells during infectious conditions or in polarization conditions [83]. In low inflammatory or steady conditions, SCFAs increase IL-10^+^ CD4^+^ T cells. Thus, they can suppress inflammatory responses when immune tolerance is needed. There have been reports about increased FoxP3^+^ Tregs induced by SCFAs [84, 85]. This function is likely to be indirect because T cells rarely express the GPCRs for SCFAs. Therefore, most of the functions of SCFAs on T cells are thought to be mediated through their metabolic and HDAC inhibition effects. In this regard, SCFAs increase the activity of mTOR [83], which supports all T cells but particularly effector T cells. The functions of cytotoxic effector T cells during viral infections and in cancers can be boosted by SCFAs [86–90]. SCFAs increase cellular metabolism in CD8^+^ T cells. Moreover, SCFAs enhanced the memory potential of activated CD8^+^ T cells and supported optimal memory recall responses. SCFAs also boost B cell differentiation into antibody producing plasma B cells [18]. Dietary fiber or SCFA deficiency leads to decreased antibody production. The production of IgG and IgA is particularly supported by SCFAs, whereas IgE production was suppressed by relatively high levels of SCFAs [91]. A caveat is that suppression of antibodies has been reported at high levels of SCFAs due to HDAC inhibition [92]. In this case, SCFAs upregulated miRNAs that silence the expression of activation-induced cytidine deaminase (AID) to suppress antibody production. Importantly, SCFAs, absorbed into B cells, can be converted to acetyl-CoA to feed the TCA cycle to increase the B cell energy level. The elevated acetyl-CoA supports fatty acid synthesis, which is required for plasma B cell generation [18]. HDAC inhibition by SCFAs also contributes to gene expression necessary for plasma B cell differentiation. For example, the expression of *Aicda*, *Xbp1*, and *Prdm1* (Blimp-1) is increased by SCFAs [18]. In contrast to IgG and IgA antibodies, the production of IgE has been reported to be suppressed by SCFAs. This appears to be correlated with suppressed production of IL-13 by CD4^+^ T cells induced by butyrate [93].

While relatively understudied, MCFAs have the potential to regulate immune cells with their high metabolic property and GPCR-activating effects. MCFAs promote the generation of less inflammatory macrophages by affecting the expression of metabolic and inflammatory genes in macrophages [94]. In this regard, MCFAs can act on intestinal epithelial cells to increase barrier function and associated host defense functions, and blunt inflammatory responses [95]. MCFAs can decrease toll-like receptor (TLR)-mediated inflammatory responses [96, 97]. They can change the composition of gut bacteria by favoring the growth of beneficial bacteria and improving barrier integrity [98, 99]. MCFAs affect microbiomes, in part, by inhibiting the growth of certain harmful microbes and strengthening intestinal anti-microbial resistance [95, 100].

LCFAs function in the immune system as membrane components, energy sources, and signaling molecules that modulate immune cell activity. They can be both pro-inflammatory and anti-inflammatory, depending on the specific fatty acid and context. For instance, some LCFAs can increase T-cell proliferation, while others, particularly omega-3 PUFAs are converted into mediators that help resolve inflammation and support antimicrobial defense. LCFAs serve as a major energy source, especially through fatty acid oxidation in mitochondria [101, 102]. LCFAs are particularly important for certain immune cells such as regulatory T cells (Tregs), memory T cells, and M2 type macrophages [103–107]. Monounsaturated LCFA and MUFAs (e.g., oleic acid) increase T-cell proliferation through incorporation into the cell membrane and promote the generation of specialized effector T cells such as IL-9-producing CD4^+^ T cells [108, 109]. The major SFA, palmitic acid, is accumulated in CD8^+^ T cells in tumors and impairs mitochondrial function [110]. Activation of GPR40 (FFA1) triggers secretion of IL-6 [111]. It is also reported that GPR40 signaling antagonizes, rather than mediates, palmitic acid-induced inflammatory responses [112]. Moreover, LCFAs activate peroxisome proliferator-activated receptors (PPARs) (Figs. 2 and 3), which influence the transcription of genes involved in inflammation and metabolism [113–115].

SFAs, such as palmitic acid and stearic acid, indirectly promote TLR2 and TLR4 activation by recruiting them to membrane rafts and greatly increase mitochondrial β-oxidation for reactive oxygen species production, leading to NLRP3 inflammasome activation in macrophages to produce TNFα, IL1β and IL-6 [116–118]. This has ramifications for low-grade inflammatory conditions associated with increased fatty acids in blood and obesity. In contrast to the proinflammatory function of LCFAs, a GPR40 agonist TAK875 suppressed the NLRP3 inflammasome by activating the sarco-endoplasmic reticulum calcium ATPase (SERCA), which lowers cytosolic calcium ion levels, and inhibits activation of NF-κB pathway and production of inflammatory cytokines [119]. In this study, TAK875 suppressed LPS-GalN-induced fulminant hepatitis in a mouse model. This suggests that GPR40 signaling does not represent the overall immune-regulatory function of LCFAs. It is also possible that the specific signaling pathways activated by GPR40 agonism can be ligand-dependent (e.g., biased agonism), leading to divergent downstream effects on inflammatory processes. Additionally, it appears that GPR40 activation can result in different effects depending on cell type. For example, GPR40 in neutrophils promotes the clearance of bacterial infections by enhancing neutrophil activity [120]. When activated by free fatty acids, GPR40 modulates functions such as superoxide production, the release of matrix metalloproteinase-9, and the expression of the integrin CD11b for cell migration. This enhances the overall ability of neutrophils to fight infection. GPR84 on macrophages has a pro-inflammatory role, promoting phagocytosis, chemotaxis, and the release of inflammatory cytokines [121]. Its activation is linked to inflammation and metabolic diseases, and it can enhance the pro-inflammatory function of macrophages and other immune cells like neutrophils and monocytes. However, GPR84 can also negatively impact immune function in other ways, such as promoting the immunosuppressive function of myeloid-derived suppressor cells (MDSCs), which can hinder anti-tumor CD8^+^ T cell immunity [122].

GPR40 is highly expressed in B cells (especially by naïve and germinal center B cells), and it plays a negative role in B cell activation and differentiation for antibody production [56]. In this regard, GPR40 suppressed BCR signaling by binding to Gαq protein. Collagen-induced arthritis (CIA) in mice was exacerbated when GPR40 was suppressed. Inhibition of GPR40 or its deficiency in B cells exacerbated the onset of CIA in mice. In humans, GPR40 is downregulated on B cells in rheumatoid patients [56]. GPR40 in macrophages primarily functions as an anti-inflammatory regulator, suppressing the secretion of pro-inflammatory cytokines (e.g, IL-6, MCP-1, and TNF-α) [123]. It achieves this in part by inhibiting signaling pathways, such as the ERK pathway, that would otherwise be activated by LPS and free fatty acids. GPR40 activation can also suppress other pro-inflammatory macrophage activities like the NLRP3 inflammasome and osteoclast differentiation [124, 125].

Anti-inflammatory or inflammation resolution functions of PUFAs are well known. As discussed earlier in this article, omega-3 fatty acids like DHA and EPA are converted into specialized pro-resolving mediators that actively reduce inflammation. These omega-3 metabolites suppress pro-inflammatory cytokine production and promote tissue repair [126]. Omega-3 fatty acids can also increase the phagocytic ability of macrophages to engulf and clear microbes [127]. Omega-3 fatty acids trigger GPR120, in part, to exert its anti-inflammatory effect [128]. GPR120 activation inhibits pro-inflammatory pathways and cytokines. GPR120 is highly expressed by dendritic cells (DCs) and alveolar macrophages in the immune system, and it promotes the induction of regulatory DCs that promote immune tolerance [129]. GPR120 can also regulate IL-10 production in T cells, which helps control colitis [130]. In contrast, the functions of omega-6 PUFA metabolites, such as, prostaglandin E2 (PGE2) and leukotriene B4 (LTB4), are largely pro-inflammatory [131].

GPR119 does not appear to directly act on immune cells but it plays a role in regulating inflammation through its effects on incretin secretion and metabolism regulation [132]. GPR119 is predominantly expressed in the gastrointestinal tract and pancreas, where it regulates the release of glucagon-like peptide-1 (GLP-1) and glucose-dependent insulinotropic polypeptide (GIP) [133]. These incretins, in turn, have anti-inflammatory properties [134].

It seems contradictory that LCFAs, particularly SFAs, are pro-inflammatory while their receptors, such as GPR40 and GPR120, are anti-inflammatory. The pro-inflammatory effects would occur when LCFAs acting directly as cellular stressors [135–137]. Excess LCFAs can overwhelm cells’ ability to process or store them safely, leading to a state of “lipotoxicity“. This triggers a cascade of direct cellular stressors, including mitochondrial dysfunction, ER stress, inflammatory responses, and cell death. SFAs, such as palmitic acid, are particularly potent stressors, while unsaturated LCFAs, such as oleic acid (a ω-9 MUFA), are often better tolerated. In contrast, the anti-inflammatory effects would occur when other fatty acids, such as MUFAs, MCFAs and PUFAs, compete with SFAs to lessen their stressor and inflammatory effects. Additionally, differential triggering GPR84, GPR40 and GPR120 may induce inflammation-repressing signaling cascades [138, 139]. It is currently unclear how the activation of GPR40 and GPR120, which are activated by both SFAs and MUFAs, induces such suppressive effects on SFA-induced inflammatory responses. While more studies are required for clear understanding, it has been reported that different fatty acids differentially bind these GPCRs for triggering distinct effects [140]. MUFAs allosterically activate Sirtuin 1 (SIRT1) to deacetylate and activate peroxisome proliferator-activated receptor gamma coactivator 1 alpha (PGC-1α) [141], which is a master transcriptional coactivator that regulates cellular energy metabolism, mitochondrial biogenesis, and adaptive thermogenesis in muscle, brain, and fat cells. PGC-1α is activated by various environmental signals, such as cold, exercise, and metabolic changes. Interestingly, MUFA-mediated activation of PGC-1α promotes hair follicle stem cell growth [142].

## Impact of SCFAs, MCFAs, and LCFAs on immunological diseases

As implied by the regulatory functions of fatty acids in regulating various immune cells, the fatty acids have significant effects on the pathogenesis of many immunological diseases. SCFA deficiencies are caused by diets low in fiber and are linked to both inflammatory and metabolic diseases, with SCFAs playing a complex role that can be beneficial or detrimental depending on the specific disease, concentration, and tissues [143]. Altered SCFA levels are associated with disorders like asthma, inflammatory diseases, autoimmune diseases, and metabolic diseases [144]. Asthma and allergic diseases are largely mediated by B cell-derived antibodies (particularly IgE), T cells (Th2), ILC2, mast cells, basophils, and eosinophils [145]. These diseases are thought to be initiated by many factors including weakened barrier function and other conditions that increase antigen transport and innate immune activation, leading to subsequent lymphocyte sensitization to allergens [146]. SCFAs negatively regulate the activities of ILC2s and mast cells [18, 42, 76, 77, 83, 84, 147]. Butyrate and propionate have been shown to increase Treg generation but decrease M2 type macrophages polarization [84, 148]. SCFAs, prebiotics or SCFA-producing microbes have been shown to attenuate airway hyperresponsiveness and inflammation [147]. The expression of pro-allergic genes and cytokines, particularly IL-4, produced by immune cells was decreased by SCFAs [76]. As a mechanism, HDAC inhibition by SCFAs seems most important for T cells, B cells, and ILC2, and activation of GPR43 and GPR109A is also involved in suppressing the inflammatory activities of epithelial cells, antigen presenting cells and ILC3 (Fig. 3) [148, 149].

SCFAs can influence the balance of regulatory and effector immune cells, potentially affecting the development of conditions such as type 1 diabetes, rheumatoid arthritis, and multiple sclerosis by modulating immune cell activity to promote immune tolerance [143]. Inflammatory bowel disease (IBD) and colon cancer are also suppressed by SCFAs, dietary fiber and SCFA-producing microbes [150–152]. Normal gut barrier integrity is key to preventing IBD, and SCFAs are crucial for maintaining the barrier by supplying an energy source and regulating gene expression in colonocytes. Additionally, the SCFA receptors GPR43 and GPR41 are important for maintaining the epithelial barrier. Accordingly, changes in SCFA levels are linked to IBD [153, 154]. A caveat is that SCFAs can increase effector T and B cell functions [18, 83], which may in certain conditions have the potential to exacerbate inflammatory activities.

MCFAs, such as caprylic acid, capric acid, and lauric acid, can disrupt pathogens’ membranes and therefore have natural antimicrobial properties that may help fight harmful bacteria, viruses, fungi, and parasites [155]. Caprylic acid has been shown to downregulate the inflammatory NF-κB signaling pathway while modulating cytokines like IL-6 and IL-8, and to suppress atherosclerosis [96, 156]. Studies have shown both positive and negative effects of MCFAs on autoimmune diseases. In experimental autoimmune encephalomyelitis (EAE), an animal model of multiple sclerosis, diets rich in specific MCFAs have been shown to worsen disease symptoms [157, 158]. These pro-inflammatory MCFAs can promote the differentiation of Th1 and Th17 cells in the gut, which then migrate to the central nervous system (CNS) and activate inflammation via the p38-MAPK signaling pathway. High levels of caproic acid and low levels of beneficial SCFAs have been found in the blood of MS patients compared to healthy individuals [159, 160]. The detrimental effects of MCFAs can be transferred via fecal transplant, suggesting that MCFAs influence MS susceptibility through microbial changes [157]. Mixed effects of MCFAs on IBD have been reported [161, 162]. The levels of MCFAs, such as octanoate, nonanoate, pentanoate, hexanoate, and heptanoate, have been found decreased in IBD patients [161]. Interestingly, dextran sodium sulfate (DSS) forms complexes with MCFAs in the intestine to induce colitis in animals [163]. It appears that MCFAs potentiate the inflammatory effects of certain toxicants.

Altered lipid metabolism, or dyslipidemia is linked to autoimmune rheumatic diseases, such as rheumatoid arthritis and systemic lupus erythematosus (SLE) [164]. In patients with SLE, elevated levels of MCFAs have been observed, along with elevated LDL and reduced levels of HDL and long-chain fatty acids [165, 166]. This suggests that lipid metabolism, including the catabolism of MCFAs, is altered in SLE. However, the increased MCFAs could be a result, not the cause, of inflammatory conditions or medications. Whatever the reasons, altered fatty acid metabolism occurs in SLE patients and this has the potential to affect the disease activity.

In animal production, the infection with pathogenic *E. coli* in chickens was decreased by dietary MCFAs potentially by killing harmful bacteria and improving immunity, beneficial gut microbes, and intestinal integrity [162]. Some studies suggest certain MCFAs, particularly the 12C-lauric acid, may have pro-inflammatory effects in the gut by stimulating the release of IL-8 and other inflammatory cytokines by potentially promoting the activation of Nod1, Nod2, and NF-κB [167]. It has been reported also in mice that MCFAs have been shown to regulate inflammation in intestinal epithelial cells by reducing IL-8 secretion [168]. Thus, the effects of MCFAs are bidirectional and the overall effects of MCFAs on gut health and microbiota balance appear to depend on context. Another factor is the amount or dosage used in nutritional and in vitro studies, where moderate levels are protective whereas high levels are often inflammatory.

In many immunological diseases, saturated LCFAs and omega-6 PUFA have generally adverse effects, whereas omega-3 PUFAs have protective effects. omega-3 and omega-6 LCFAs are precursors to different types of lipid mediators. Omega-6 PUFAs are converted into pro-inflammatory mediators, such as prostaglandins, while omega-3 PUFAs produce anti-inflammatory specialized pro-resolving mediators (SPMs) and others that decrease inflammation. In this regard, a diet low in omega-3 PUFAs and high in omega-6 PUFAs (e.g., linoleic acid) worsened EAE activity in mice [169, 170]. Omega-3 PUFAs, such as EPA and DHA, ameliorated the inflammation in rheumatoid arthritis [171]. While omega-3s were largely anti-inflammatory and beneficial on IBD, some omega-6s promoted intestinal inflammation [172, 173]. The balance of dietary omega-6 and omega-3 PUFAs also affects asthma severity [174]. An imbalanced ratio is associated with poorer asthma control. Omega-3 PUFAs supplementation has been effective in managing some forms of asthma [174]. Omega-3 supplementation during pregnancy and infancy prevented atopic dermatitis or decreased its severity [175]. Moreover, early life exposure to PUFAs has been linked to a lower risk of food allergy development [176]. Additionally, omega-3 PUFAs had anti-inflammatory and protective effects on atherosclerosis, whereas SFAs increased the formation of foam cells and plaques [177]. LCFAs in the tumor microenvironment can influence anti-tumor immunity, affecting the efficacy of immunotherapy. Some LCFAs can promote tumor growth by fueling cancer cell metabolism and promoting an immunosuppressive environment [178]. In a study, it has been observed that a high level of linoleic acid (an omega-6 PUFA) impaired anti-tumor T cell function by promoting apoptosis, inhibiting TNFα production, and inducing mitochondrial dysfunction [179]. Another study reported that an in vitro treatment of CD8^+^ T cells with linoleic acid or oleic acid (a MUFA) decreased T cell exhaustion and stimulated a memory-like phenotype [180]. Thus, T cell function is differentially regulated by the LCFA types.

Fatty acids play key roles in regulating metabolic diseases. SCFAs suppress obesity and metabolic syndrome by regulating energy intake and expenditure, potentially by decreasing appetite [181–185]. Similarly, SCFAs protect against diabetes by increasing glycogen storage and decreasing glycolysis to control blood glucose levels [186]. The SCFA receptor GPR43 functions to suppress insulin-mediated fat accumulation [43]. Insufficient SCFA levels are caused by low-fiber diet intake or other reasons such as microbial dysbiosis and various health issues. Inadequate intake of dietary fiber, microbial dysbiosis, and decreased SCFA levels are linked to insulin resistance and an increased risk of developing diabetes [187–191]. SCFAs decreased autoimmune CD4^+^ and CD8^+^ T cell but increased Treg activity in experimental models of type I diabetes [192]. Also, elevating the level of SCFAs changed microbiome and microbial metabolites to enhance intestinal homeostasis in humanized gnotobiotic type I diabetes mice [193]. Despite the presence of animal model-dependent variations in responses, SCFA treatments strengthened the gut barrier, decreased inflammation, and suppressed non-alcoholic fatty liver disease [14, 194]. The underlying mechanisms for these functions include suppressed lipogenesis and lowered cholesterol as well as SCFA-induced incretin secretion. In this regard, SCFAs trigger GPR41, GPR43, and Olfr78 on intestinal enteroendocrine cells to secrete glucagon-like peptide-1 (GLP-1) and peptide YY (PYY) [44, 195–199]. SCFAs also induce leptin secretion from adipocytes by activating GPR41 and GPR43 [46, 47] Overall, SCFAs increase energy expenditure, fatty acid oxidation and insulin sensitivity [200, 201]. Moreover, SCFAs regulate inflammatory cytokine production in a variety of immune cells, such as neutrophils, macrophages, ILCs, T cells, B cells, and Tregs to indirectly affect metabolic diseases [202].

Unlike LCFAs, consuming MCFAs increases the overall energy expenditure, contributing to a lower overall fat mass. In skeletal muscle and brown adipose tissue, MCFAs increase mitochondrial capacity for fatty acid oxidation and reduce oxidative stress [203]. This supports more efficient energy burning. Rapid oxidation of MCFAs leads to the production of ketone bodies, such as $\beta$-hydroxy butyrate [3]. Ketones can be used by the brain and other tissues for energy, providing an alternative fuel source that influences appetite/food intake and energy balance [204]. Similar to SCFAs, MCFA consumption triggers the release of satiety-related hormones like leptin and peptide YY (PYY) [205]. This effect prolongs the feeling of satiety to reduce food intake. MCFAs help prevent and reverse the buildup of fat in the liver (i.e., hepatic steatosis) [206]. However, “Western diet,” which is rich in SFAs and omega-6 PUFAs, promotes obesity and is linked to systemic inflammation and impaired immune responses [207]. This drives inflammation in various tissues, including the liver and adipose tissue. LCFAs promote metabolic diseases and obesity in part through disrupted energy balance, insulin resistance, chronic inflammation, and altered gene expression [208]. The specific effects depend on the type of LCFA, with some PUFAs having different effects than SFAs. In metabolic diseases, some MCFAs (e.g., capric acid, C10:0) and SFAs (e.g., palmitic acid and stearic acid) can promote pro-inflammatory immune responses by shifting T cell functions from immune-suppressive or regulatory towards pro-inflammatory types [209–211]. This is largely the opposite effect of SCFAs. SFAs can exacerbate autoimmune diseases, in part, by promoting the migration of pro-inflammatory T cells to affected tissues [160, 212]. SFAs also promote an imbalance in gut microbes and their metabolism [213–215], thereby further amplifying their proinflammatory effects in the body. High consumption of SFAs decreased microbial diversity and the abundance of butyrate-producing microbes, adversely affecting liver metabolic status in a human cohort [216].

## Concluding remarks

Fatty acids manifest various beneficial and harmful effects on the immune system. SFAs, preferred for energy storage in adipose tissues, are implicated in obesity, lipotoxicity and inflammation. Excessive consumption of SFAs can compromise barrier functions and activate various immune cells that mediate inflammatory responses. In contrast, MUFAs support barrier function and largely suppress inflammatory immune cells. These differences are thought to be due to differences in their physical properties that affect membrane signaling, including TLR4, NF-κB, NLRP3 inflammasome, and GPR120. The largely specific expression of GPR40 by B cells and GPR120 by DCs in the immune system would have selective effects on antibody responses and T cell immunity among others (Fig. 3). Moreover, their metabolic properties in cells and the body contribute to their overall function by generating subsequent metabolites, particularly acetyl-CoA, which trigger downstream effects mediated by additional receptors. Moreover, omega-3 and omega-6 PUFAs serve as precursors for distinct immune-regulatory metabolites with different roles in the immune system. The balance between omega-3 and omega-6 PUFAs has been demonstrated to be important in prevention vs. induction of inflammatory responses and diseases. Protective LCFAs, such as omega-3 and MUFAs, compete with SFAs to antagonize their inflammatory functions. MCFAs are efficient in their conversion to acetyl-CoA, rather than stored as fat, to fuel cells and support immune cell differentiation for host defense at tissue barriers. Inflammatory diseases occur often following inadequate functioning of barrier immunity. It is also important to note that glucose is converted to palmitic acid (a SFA) through a process called de novo lipogenesis in liver, adipocytes, and macrophages but less likely to protective PUFAs and MCFAs. SCFAs are readily metabolized to make acetyl-CoA and activate several GPCRs on epithelial cells, macrophages, neutrophils, ILCs, and DCs, which together control both innate and adaptive immunity. They additionally control the activities of intracellular targets such as HDACs and HATs to facilitate gene expression in most cell types. The immunological effects of fatty acids are also determined by their availability, tissue environments, and target cell types.

An important factor that has not been discussed in depth in this article is their differential effects on the gut microbiome for additional regulatory effects. In short, SFAs induce microbial dysbiosis, leading to decreased diversity and abnormal shifts in microbial groups (e.g., decreased beneficial *Firmicutes* but increased *Enterobacteriaceae* and *Bilophila*) [217]. In contrast, SCFAs and, to a large degree, MUFAs and MCFAs, can increase beneficial microbes (e.g., *Bacteroidetes*, *Ruminococcaceae*, and *Bifidobacterium*) in the gut [218]. While the exact mechanisms for this regulation remain unclear, direct and indirect regulations through the immune system are likely to be involved. While dissection of the diverse functions of fatty acids in terms of specific molecular targets and pathways is a daunting task, further research with genetically engineered cell and animal models and specific agonists and antagonists that can separate their functions mediated by differential GPCR agonism, metabolism, microbial regulation, and membrane homeostasis would yield further insights regarding the immune regulatory functions of the fatty acids.

## References

[1] Kim CH. Control of lymphocyte functions by gut microbiota-derived short-chain fatty acids. Cell Mol Immunol. 2021;18:1161–71. 33850311 10.1038/s41423-020-00625-0 PMC8093302

[2] Bolognini D, Dedeo D, Milligan G. Metabolic and inflammatory functions of short-chain fatty acid receptors. Curr Opin Endocr Metab Res. 2021;16:1–9. 32835130 10.1016/j.coemr.2020.06.005 PMC7332907

[3] Odle J. New insights into the utilization of medium-chain triglycerides by the neonate: observations from a piglet model. J Nutr. 1997;127:1061–7. 9187618 10.1093/jn/127.6.1061

[4] Jia M, Zhang Y, Gao Y, Ma X. Effects of medium chain fatty acids on intestinal health of monogastric animals. Curr Protein Pept Sci. 2020;21:777–84. 31889482 10.2174/1389203721666191231145901

[5] He Q, Chen Y, Wang Z, He H, Yu P. Cellular uptake, metabolism and sensing of long-chain fatty acids. Front Biosci (Landmark Ed). 2023;28:10. 36722264 10.31083/j.fbl2801010

[6] Nakamura MT, Yudell BE, Loor JJ. Regulation of energy metabolism by long-chain fatty acids. Prog Lipid Res. 2014;53:124–44. 24362249 10.1016/j.plipres.2013.12.001

[7] Mashek DG, Coleman RA. Cellular fatty acid uptake: the contribution of metabolism. Curr Opin Lipido. 2006;17:274–8. 10.1097/01.mol.0000226119.20307.2b 16680032

[8] Calder PC. Fatty acids and inflammation: the cutting edge between food and pharma. Eur J Pharm. 2011;668:S50–58. 10.1016/j.ejphar.2011.05.085 21816146

[9] Calder PC. Functional roles of fatty acids and their effects on human health. JPEN J Parenter Enter Nutr. 2015;39:18S–32S. 10.1177/0148607115595980 26177664

[10] Macfarlane S, Macfarlane GT. Regulation of short-chain fatty acid production. Proc Nutr Soc. 2003;62:67–72. 12740060 10.1079/PNS2002207

[11] Louis P, Hold GL, Flint HJ. The gut microbiota, bacterial metabolites and colorectal cancer. Nat Rev Microbiol. 2014;12:661–72. 25198138 10.1038/nrmicro3344

[12] Morrison DJ, Preston T. Formation of short chain fatty acids by the gut microbiota and their impact on human metabolism. Gut Microbes. 2016;7:189–200. 26963409 10.1080/19490976.2015.1134082 PMC4939913

[13] Koh A, De Vadder F, Kovatcheva-Datchary P, Backhed F. From Dietary fiber to host physiology: short-chain fatty acids as key bacterial metabolites. Cell. 2016;165:1332–45. 27259147 10.1016/j.cell.2016.05.041

[14] Pant K, Venugopal SK, Lorenzo Pisarello MJ, Gradilone SA. The Role of gut microbiome-derived short-chain fatty acid butyrate in hepatobiliary diseases. Am J Pathol. 2023;193:1455–67. 37422149 10.1016/j.ajpath.2023.06.007 PMC10548274

[15] Pluznick JL. Microbial short-chain fatty acids and blood pressure regulation. Curr Hypertens Rep. 2017;19:25. 28315048 10.1007/s11906-017-0722-5 PMC5584783

[16] Takeuchi T, Nakanishi Y, Ohno H. Microbial metabolites and gut immunology. Annu Rev Immunol. 2024;42:153–78. 38941602 10.1146/annurev-immunol-090222-102035

[17] Hoverstad T, Bohmer T, Fausa O. Absorption of short-chain fatty acids from the human colon measured by the 14CO2 breath test. Scand J Gastroenterol. 1982;17:373–8. 6813956 10.3109/00365528209182070

[18] Kim M, Qie Y, Park J, Kim CH. Gut microbial metabolites fuel host antibody responses. Cell Host Microbe. 2016;20:202–14. 27476413 10.1016/j.chom.2016.07.001 PMC4982788

[19] Zambell KL, Fitch MD, Fleming SE. Acetate and butyrate are the major substrates for de novo lipogenesis in rat colonic epithelial cells. J Nutr. 2003;133:3509–15. 14608066 10.1093/jn/133.11.3509

[20] Tietz A, Ochoa S. Metabolism of propionic acid in animal tissues. V. Purification and properties of propionyl carboxylase. J Biol Chem. 1959;234:1394–1400. 13654385

[21] Arnold PK, Finley LWS. Regulation and function of the mammalian tricarboxylic acid cycle. J Biol Chem. 2023;299:102838. 36581208 10.1016/j.jbc.2022.102838 PMC9871338

[22] Candido EP, Reeves R, Davie JR. Sodium butyrate inhibits histone deacetylation in cultured cells. Cell. 1978;14:105–13. 667927 10.1016/0092-8674(78)90305-7

[23] Thomas SP, Denu JM. Short-chain fatty acids activate acetyltransferase p300. Elife. 2021;10:1–23. 10.7554/eLife.72171 PMC8585482 34677127

[24] Lee C, Kim BG, Kim JH, Chun J, Im JP, Kim JS. Sodium butyrate inhibits the NF-kappa B signaling pathway and histone deacetylation, and attenuates experimental colitis in an IL-10 independent manner. Int Immunopharmacol. 2017;51:47–56. 28802151 10.1016/j.intimp.2017.07.023

[25] Inan MS, Rasoulpour RJ, Yin L, Hubbard AK, Rosenberg DW, Giardina C. The luminal short-chain fatty acid butyrate modulates NF-kappaB activity in a human colonic epithelial cell line. Gastroenterology. 2000;118:724–34. 10734024 10.1016/s0016-5085(00)70142-9

[26] Macia L, Tan J, Vieira AT, Leach K, Stanley D, Luong S, et al. Metabolite-sensing receptors GPR43 and GPR109A facilitate dietary fibre-induced gut homeostasis through regulation of the inflammasome. Nat Commun. 2015;6:6734. 25828455 10.1038/ncomms7734

[27] Tsugawa H, Kabe Y, Kanai A, Sugiura Y, Hida S, Taniguchi S, et al. Short-chain fatty acids bind to apoptosis-associated speck-like protein to activate inflammasome complex to prevent Salmonella infection. PLoS Biol. 2020;18:e3000813. 32991574 10.1371/journal.pbio.3000813 PMC7524008

[28] Hamosh M. Lingual and gastric lipases. Nutrition. 1990;6:421–8. 2134569

[29] Bach AC, Babayan VK. Medium-chain triglycerides: an update. Am J Clin Nutr. 1982;36:950–62. 6814231 10.1093/ajcn/36.5.950

[30] Stahl A, Hirsch DJ, Gimeno RE, Punreddy S, Ge P, Watson N, et al. Identification of the major intestinal fatty acid transport protein. Mol Cell. 1999;4:299–308. 10518211 10.1016/s1097-2765(00)80332-9

[31] Masson CJ, Plat J, Mensink RP, Namiot A, Kisielewski W, Namiot Z, et al. Fatty acid- and cholesterol transporter protein expression along the human intestinal tract. PLoS One. 2010;5:e10380. 20454462 10.1371/journal.pone.0010380 PMC2861623

[32] Kaur N, Chugh V, Gupta AK. Essential fatty acids as functional components of foods- a review. J Food Sci Technol. 2014;51:2289–303. 25328170 10.1007/s13197-012-0677-0 PMC4190204

[33] Ormseth MJ, Swift LL, Fazio S, Linton MF, Raggi P, Solus JF, et al. Free fatty acids are associated with metabolic syndrome and insulin resistance but not inflammation in systemic lupus erythematosus. Lupus. 2013;22:26–33. 23060481 10.1177/0961203312462756 PMC3684362

[34] van der Vusse GJ. Albumin as fatty acid transporter. Drug Metab Pharmacokinet. 2009;24:300–7. 19745557 10.2133/dmpk.24.300

[35] Philp NJ, Yoon H, Grollman EF. Monocarboxylate transporter MCT1 is located in the apical membrane and MCT3 in the basal membrane of rat RPE. Am J Physiol. 1998;274:R1824–1828. 9841555 10.1152/ajpregu.1998.274.6.R1824

[36] Ritzhaupt A, Wood IS, Ellis A, Hosie KB, Shirazi-Beechey SP. Identification and characterization of a monocarboxylate transporter (MCT1) in pig and human colon: its potential to transport L-lactate as well as butyrate. J Physiol. 1998;513:719–32. 9824713 10.1111/j.1469-7793.1998.719ba.x PMC2231331

[37] Martin PM, Gopal E, Ananth S, Zhuang L, Itagaki S, Prasad BM, et al. Identity of SMCT1 (SLC5A8) as a neuron-specific Na + -coupled transporter for active uptake of L-lactate and ketone bodies in the brain. J Neurochem. 2006;98:279–88. 16805814 10.1111/j.1471-4159.2006.03878.x

[38] Nassir F, Wilson B, Han X, Gross RW, Abumrad NA. CD36 is important for fatty acid and cholesterol uptake by the proximal but not distal intestine. J Biol Chem. 2007;282:19493–501. 17507371 10.1074/jbc.M703330200

[39] Kurtz DM, Rinaldo P, Rhead WJ, Tian L, Millington DS, Vockley J, et al. Targeted disruption of mouse long-chain acyl-CoA dehydrogenase gene reveals crucial roles for fatty acid oxidation. Proc Natl Acad Sci USA. 1998;95:15592–7. 9861014 10.1073/pnas.95.26.15592 PMC28088

[40] Sanders FW, Griffin JL. De novo lipogenesis in the liver in health and disease: more than just a shunting yard for glucose. Biol Rev Camb Philos Soc. 2016;91:452–68. 25740151 10.1111/brv.12178 PMC4832395

[41] Sacks FM, Lichtenstein AH, Wu JHY, Appel LJ, Creager MA, Kris-Etherton PM, et al. Dietary fats and cardiovascular disease: a presidential advisory from the American Heart Association. Circulation. 2017;136:e1–e23. 28620111 10.1161/CIR.0000000000000510

[42] Kim MH, Kang SG, Park JH, Yanagisawa M, Kim CH. Short-chain fatty acids activate GPR41 and GPR43 on intestinal epithelial cells to promote inflammatory responses in mice. Gastroenterology. 2013;145:396–406.e391-310. 23665276 10.1053/j.gastro.2013.04.056

[43] Kimura I, Ozawa K, Inoue D, Imamura T, Kimura K, Maeda T, et al. The gut microbiota suppresses insulin-mediated fat accumulation via the short-chain fatty acid receptor GPR43. Nat Commun. 2013;4:1829. 23652017 10.1038/ncomms2852 PMC3674247

[44] Nohr MK, Pedersen MH, Gille A, Egerod KL, Engelstoft MS, Husted AS, et al. GPR41/FFAR3 and GPR43/FFAR2 as cosensors for short-chain fatty acids in enteroendocrine cells vs FFAR3 in enteric neurons and FFAR2 in enteric leukocytes. Endocrinology. 2013;154:3552–64. 23885020 10.1210/en.2013-1142

[45] Le Poul E, Loison C, Struyf S, Springael JY, Lannoy V, Decobecq ME, et al. Functional characterization of human receptors for short chain fatty acids and their role in polymorphonuclear cell activation. J Biol Chem. 2003;278:25481–9. 12711604 10.1074/jbc.M301403200

[46] Xiong Y, Miyamoto N, Shibata K, Valasek MA, Motoike T, Kedzierski RM, et al. Short-chain fatty acids stimulate leptin production in adipocytes through the G protein-coupled receptor GPR41. Proc Natl Acad Sci USA. 2004;101:1045–50. 14722361 10.1073/pnas.2637002100 PMC327148

[47] Zaibi MS, Stocker CJ, O'Dowd J, Davies A, Bellahcene M, Cawthorne MA, et al. Roles of GPR41 and GPR43 in leptin secretory responses of murine adipocytes to short chain fatty acids. FEBS Lett. 2010;584:2381–6. 20399779 10.1016/j.febslet.2010.04.027

[48] Chun E, Lavoie S, Fonseca-Pereira D, Bae S, Michaud M, Hoveyda HR, et al. Metabolite-sensing receptor Ffar2 regulates colonic group 3 innate lymphoid cells and gut immunity. Immunity. 2019;51:871–884.e876. 31628054 10.1016/j.immuni.2019.09.014 PMC6901086

[49] Singh N, Gurav A, Sivaprakasam S, Brady E, Padia R, Shi H, et al. Activation of Gpr109a, receptor for niacin and the commensal metabolite butyrate, suppresses colonic inflammation and carcinogenesis. Immunity. 2014;40:128–39. 24412617 10.1016/j.immuni.2013.12.007 PMC4305274

[50] Secor JD, Fligor SC, Tsikis ST, Yu LJ, Puder M. Free fatty acid receptors as mediators and therapeutic targets in liver disease. Front Physiol. 2021;12:656441. 33897464 10.3389/fphys.2021.656441 PMC8058363

[51] Wang J, Wu X, Simonavicius N, Tian H, Ling L. Medium-chain fatty acids as ligands for orphan G protein-coupled receptor GPR84. J Biol Chem. 2006;281:34457–64. 16966319 10.1074/jbc.M608019200

[52] Schulze AS, Kleinau G, Krakowsky R, Rochmann D, Das R, Worth CL, et al. Evolutionary analyses reveal immune cell receptor GPR84 as a conserved receptor for bacteria-derived molecules. iScience. 2022;25:105087. 36164652 10.1016/j.isci.2022.105087 PMC9508565

[53] Overton HA, Babbs AJ, Doel SM, Fyfe MC, Gardner LS, Griffin G, et al. Deorphanization of a G protein-coupled receptor for oleoylethanolamide and its use in the discovery of small-molecule hypophagic agents. Cell Metab. 2006;3:167–75. 16517404 10.1016/j.cmet.2006.02.004

[54] Kogure R, Toyama K, Hiyamuta S, Kojima I, Takeda S. 5-Hydroxy-eicosapentaenoic acid is an endogenous GPR119 agonist and enhances glucose-dependent insulin secretion. Biochem Biophys Res Commun. 2011;416:58–63. 22079287 10.1016/j.bbrc.2011.10.141

[55] Hansen KB, Rosenkilde MM, Knop FK, Wellner N, Diep TA, Rehfeld JF, et al. 2-Oleoyl glycerol is a GPR119 agonist and signals GLP-1 release in humans. J Clin Endocrinol Metab. 2011;96:E1409–1417. 21778222 10.1210/jc.2011-0647

[56] Li A, Wang X, Li J, Li X, Wang J, Liu Y, et al. Critical role of G protein-coupled receptor 40 in B cell response and the pathogenesis of rheumatoid arthritis in mice and patients. Cell Rep. 2024;43:114858. 39392754 10.1016/j.celrep.2024.114858

[57] Aktar R, Rondinelli S, Peiris M. GPR84 in physiology: many functions in many tissues. Br J Pharm. 2024;181:1524–35. 10.1111/bph.16206 37533166

[58] Falomir-Lockhart LJ, Cavazzutti GF, Gimenez E, Toscani AM. Fatty acid signaling mechanisms in neural cells: fatty acid receptors. Front Cell Neurosci. 2019;13:162. 31105530 10.3389/fncel.2019.00162 PMC6491900

[59] Zhang X, Guseinov AA, Jenkins L, Li K, Tikhonova IG, Milligan G, et al. Structural basis for the ligand recognition and signaling of free fatty acid receptors. Sci Adv. 2024;10:eadj2384. 38198545 10.1126/sciadv.adj2384 PMC10780892

[60] Glukhova A, Draper-Joyce CJ, Sunahara RK, Christopoulos A, Wootten D, Sexton PM. Rules of engagement: GPCRs and G proteins. ACS Pharm Transl Sci. 2018;1:73–83. 10.1021/acsptsci.8b00026 PMC7089011 32219204

[61] Sepahi A, Liu Q, Friesen L, Kim CH. Dietary fiber metabolites regulate innate lymphoid cell responses. Mucosal Immunol. 2021;14:317–30. 32541842 10.1038/s41385-020-0312-8 PMC7736174

[62] Kotarsky K, Nilsson NE, Flodgren E, Owman C, Olde B. A human cell surface receptor activated by free fatty acids and thiazolidinedione drugs. Biochem Biophys Res Commun. 2003;301:406–10. 12565875 10.1016/s0006-291x(02)03064-4

[63] Fujiwara K, Maekawa F, Yada T. Oleic acid interacts with GPR40 to induce Ca2+ signaling in rat islet beta-cells: mediation by PLC and L-type Ca2+ channel and link to insulin release. Am J Physiol Endocrinol Metab. 2005;289:E670–677. 15914509 10.1152/ajpendo.00035.2005

[64] Morgan NG, Dhayal S. G-protein coupled receptors mediating long chain fatty acid signalling in the pancreatic beta-cell. Biochem Pharm. 2009;78:1419–27. 19660440 10.1016/j.bcp.2009.07.020

[65] Soga T, Ohishi T, Matsui T, Saito T, Matsumoto M, Takasaki J, et al. Lysophosphatidylcholine enhances glucose-dependent insulin secretion via an orphan G-protein-coupled receptor. Biochem Biophys Res Commun. 2005;326:744–51. 15607732 10.1016/j.bbrc.2004.11.120

[66] Hirasawa A, Tsumaya K, Awaji T, Katsuma S, Adachi T, Yamada M, et al. Free fatty acids regulate gut incretin glucagon-like peptide-1 secretion through GPR120. Nat Med. 2005;11:90–94. 15619630 10.1038/nm1168

[67] Lenaz G. Lipid fluidity and membrane protein dynamics. Biosci Rep. 1987;7:823–37. 3329533 10.1007/BF01119473

[68] Surette ME. The science behind dietary omega-3 fatty acids. CMAJ. 2008;178:177–80. 18195292 10.1503/cmaj.071356 PMC2174995

[69] Wen H, Gris D, Lei Y, Jha S, Zhang L, Huang MT, et al. Fatty acid-induced NLRP3-ASC inflammasome activation interferes with insulin signaling. Nat Immunol. 2011;12:408–15. 21478880 10.1038/ni.2022 PMC4090391

[70] Flamment M, Kammoun HL, Hainault I, Ferre P, Foufelle F. Endoplasmic reticulum stress: a new actor in the development of hepatic steatosis. Curr Opin Lipido. 2010;21:239–46. 10.1097/MOL.0b013e3283395e5c 20463471

[71] Yang X, Haghiac M, Glazebrook P, Minium J, Catalano PM, Hauguel-de Mouzon S. Saturated fatty acids enhance TLR4 immune pathways in human trophoblasts. Hum Reprod. 2015;30:2152–9. 26202921 10.1093/humrep/dev173 PMC4635648

[72] Le Faouder P, Baillif V, Spreadbury I, Motta JP, Rousset P, Chene G, et al. LC-MS/MS method for rapid and concomitant quantification of pro-inflammatory and pro-resolving polyunsaturated fatty acid metabolites. J Chromatogr B Anal Technol Biomed Life Sci. 2013;932:123–33. 10.1016/j.jchromb.2013.06.014 23831705

[73] Vinolo MA, Ferguson GJ, Kulkarni S, Damoulakis G, Anderson K, Bohlooly YM, et al. SCFAs induce mouse neutrophil chemotaxis through the GPR43 receptor. PLoS One. 2011;6:e21205. 21698257 10.1371/journal.pone.0021205 PMC3115979

[74] Sina C, Gavrilova O, Forster M, Till A, Derer S, Hildebrand F, et al. G protein-coupled receptor 43 is essential for neutrophil recruitment during intestinal inflammation. J Immunol. 2009;183:7514–22. 19917676 10.4049/jimmunol.0900063

[75] Maslowski KM, Vieira AT, Ng A, Kranich J, Sierro F, Yu D, et al. Regulation of inflammatory responses by gut microbiota and chemoattractant receptor GPR43. Nature. 2009;461:1282–6. 19865172 10.1038/nature08530 PMC3256734

[76] Lewis G, Wang B, Shafiei Jahani P, Hurrell BP, Banie H, Aleman Muench GR, et al. Dietary fiber-induced microbial short chain fatty acids suppress ILC2-dependent airway inflammation. Front Immunol. 2019;10:2051. 31620118 10.3389/fimmu.2019.02051 PMC6760365

[77] Thio CL, Chi PY, Lai AC, Chang YJ. Regulation of type 2 innate lymphoid cell-dependent airway hyperreactivity by butyrate. J Allergy Clin Immunol. 2018;142:1867–1883.e1812. 29522844 10.1016/j.jaci.2018.02.032

[78] Schulthess J, Pandey S, Capitani M, Rue-Albrecht KC, Arnold I, Franchini F, et al. The short chain fatty acid butyrate imprints an antimicrobial program in macrophages. Immunity. 2019;50:432–445.e437. 30683619 10.1016/j.immuni.2018.12.018 PMC6382411

[79] Galvao I, Tavares LP, Correa RO, Fachi JL, Rocha VM, Rungue M, et al. The metabolic sensor GPR43 receptor plays a role in the control of Klebsiella pneumoniae infection in the lung. Front Immunol. 2018;9:142. 29515566 10.3389/fimmu.2018.00142 PMC5826235

[80] Chang PV, Hao L, Offermanns S, Medzhitov R. The microbial metabolite butyrate regulates intestinal macrophage function via histone deacetylase inhibition. Proc Natl Acad Sci USA. 2014;111:2247–52. 24390544 10.1073/pnas.1322269111 PMC3926023

[81] Wu L, Seon GM, Kim Y, Choi SH, Vo QC, Yang HC. Enhancing effect of sodium butyrate on phosphatidylserine-liposome-induced macrophage polarization. Inflamm Res. 2022;71:641–52. 35347345 10.1007/s00011-022-01563-5

[82] Kaisar MMM, Pelgrom LR, van der Ham AJ, Yazdanbakhsh M, Everts B. Butyrate conditions human dendritic cells to prime type 1 regulatory T cells via both histone deacetylase inhibition and G protein-coupled receptor 109A signaling. Front Immunol. 2017;8:1429. 29163504 10.3389/fimmu.2017.01429 PMC5670331

[83] Park J, Kim M, Kang S, Jannasch A, Cooper B, Patterson J, et al. Short-chain fatty acids induce both effector and regulatory T cells by suppression of histone deacetylases and regulation of the mTOR–S6K pathway. Mucosal Immunol. 2015;8:80–93. 24917457 10.1038/mi.2014.44 PMC4263689

[84] Arpaia N, Campbell C, Fan X, Dikiy S, van der Veeken J, deRoos P, et al. Metabolites produced by commensal bacteria promote peripheral regulatory T-cell generation. Nature. 2013;504:451–5. 24226773 10.1038/nature12726 PMC3869884

[85] Bhaskaran N, Quigley C, Paw C, Butala S, Schneider E, Pandiyan P. Role of short chain fatty acids in controlling T(regs) and immunopathology during mucosal infection. Front Microbiol. 2018;9:1995. 30197637 10.3389/fmicb.2018.01995 PMC6117408

[86] Trompette A, Gollwitzer ES, Pattaroni C, Lopez-Mejia IC, Riva E, Pernot J, et al. Dietary fiber confers protection against flu by shaping Ly6c(-) patrolling monocyte hematopoiesis and CD8( + ) T cell metabolism. Immunity. 2018;48:992–1005.e1008. 29768180 10.1016/j.immuni.2018.04.022

[87] Bachem A, Makhlouf C, Binger KJ, de Souza DP, Tull D, Hochheiser K, et al. Microbiota-derived short-chain fatty acids promote the memory potential of antigen-activated CD8( + ) T cells. Immunity. 2019;51:285–297.e285. 31272808 10.1016/j.immuni.2019.06.002

[88] Lee AR, Wilson KR, Clarke M, Engel S, Tscharke DC, Gebhardt T, et al. GPR41 and GPR43 regulate CD8( + ) T cell priming during herpes simplex virus type 1 infection. Front Immunol. 2024;15:1332588. 38524121 10.3389/fimmu.2024.1332588 PMC10957577

[89] Yu X, Ou J, Wang L, Li Z, Ren Y, Xie L, et al. Gut microbiota modulate CD8( + ) T cell immunity in gastric cancer through Butyrate/GPR109A/HOPX. Gut Microbes. 2024;16:2307542. 38319728 10.1080/19490976.2024.2307542 PMC10854374

[90] Gaskarth DA, Fan S, Highton AJ, Kemp RA. The microbial metabolite butyrate enhances the effector and memory functions of murine CD8 + T cells and improves anti-tumor activity. Front Med (Lausanne). 2025;12:1577906. 40630475 10.3389/fmed.2025.1577906 PMC12234554

[91] Nagata K, Ando D, Ashikari T, Ito K, Miura R, Fujigaki I, et al. Butyrate, valerate, and niacin ameliorate anaphylaxis by suppressing IgE-dependent mast cell activation: roles of GPR109A, PGE2, and epigenetic regulation. J Immunol. 2024;212:771–84. 38197634 10.4049/jimmunol.2300188

[92] White CA, Pone EJ, Lam T, Tat C, Hayama KL, Li G, et al. Histone deacetylase inhibitors upregulate B cell microRNAs that silence AID and Blimp-1 expression for epigenetic modulation of antibody and autoantibody responses. J Immunol. 2014;193:5933–50. 25392531 10.4049/jimmunol.1401702 PMC4258531

[93] Yu B, Pei C, Peng W, Zheng Y, Fu Y, Wang X, et al. Microbiota-derived butyrate alleviates asthma via inhibiting Tfh13-mediated IgE production. Signal Transduct Target Ther. 2025;10:181. 40473603 10.1038/s41392-025-02263-2 PMC12141656

[94] Gaete PV, Nieves-Barreto LD, Guatibonza-Garcia V, Losada-Barragan M, Vargas-Sanchez K, Mendivil CO. Medium-chain fatty acids modify macrophage expression of metabolic and inflammatory genes in a PPAR beta/delta-dependent manner. Sci Rep. 2023;13:11573. 37463952 10.1038/s41598-023-38700-x PMC10353988

[95] Zentek J, Buchheit-Renko S, Ferrara F, Vahjen W, Van Kessel AG, Pieper R. Nutritional and physiological role of medium-chain triglycerides and medium-chain fatty acids in piglets. Anim Health Res Rev. 2011;12:83–93. 21676342 10.1017/S1466252311000089

[96] Zhang X, Xue C, Xu Q, Zhang Y, Li H, Li F, et al. Caprylic acid suppresses inflammation via TLR4/NF-kappaB signaling and improves atherosclerosis in ApoE-deficient mice. Nutr Metab (Lond). 2019;16:40. 31182969 10.1186/s12986-019-0359-2 PMC6555760

[97] Kono H, Fujii H, Asakawa M, Yamamoto M, Matsuda M, Maki A, et al. Protective effects of medium-chain triglycerides on the liver and gut in rats administered endotoxin. Ann Surg. 2003;237:246–55. 12560783 10.1097/01.SLA.0000048450.44868.B1 PMC1522134

[98] Hu Z, Liu L, Guo F, Huang J, Qiao J, Bi R, et al. Dietary supplemental coated essential oils and organic acids mixture improves growth performance and gut health along with reduces Salmonella load of broiler chickens infected with Salmonella Enteritidis. J Anim Sci Biotechnol. 2023;14:95. 37391807 10.1186/s40104-023-00889-2 PMC10314490

[99] Fan Z, Lei L, Wu X, Xing R, Du P, Wang Z, et al. Dietary fatty acids promote gut health in weaned piglets by regulating gut microbiota and immune function. Front Microbiol. 2025;16:1558588. 40270814 10.3389/fmicb.2025.1558588 PMC12014538

[100] Skrivanova E, Molatova Z, Skrivanova V, Marounek M. Inhibitory activity of rabbit milk and medium-chain fatty acids against enteropathogenic Escherichia coli O128. Vet Microbiol. 2009;135:358–62. 19019572 10.1016/j.vetmic.2008.09.083

[101] Angajala A, Lim S, Phillips JB, Kim JH, Yates C, You Z, et al. Diverse roles of mitochondria in immune responses: novel insights into immuno-metabolism. Front Immunol. 2018;9:1605. 30050539 10.3389/fimmu.2018.01605 PMC6052888

[102] Longo N, Frigeni M, Pasquali M. Carnitine transport and fatty acid oxidation. Biochim Biophys Acta. 2016;1863:2422–35. 26828774 10.1016/j.bbamcr.2016.01.023 PMC4967041

[103] Pompura, SL, Wagner, A, Kitz, A, LaPerche, J, Yosef, N, Dominguez-Villar, M, et al. Oleic acid restores suppressive defects in tissue-resident FOXP3 Tregs from patients with multiple sclerosis. J Clin Invest. 2021;131:1–15. 10.1172/JCI138519 PMC7810477 33170805

[104] O’Sullivan D, van der Windt GJ, Huang SC, Curtis JD, Chang CH, Buck MD, et al. Memory CD8( + ) T cells use cell-intrinsic lipolysis to support the metabolic programming necessary for development. Immunity. 2014;41:75–88. 25001241 10.1016/j.immuni.2014.06.005 PMC4120664

[105] Pan Y, Tian T, Park CO, Lofftus SY, Mei S, Liu X, et al. Survival of tissue-resident memory T cells requires exogenous lipid uptake and metabolism. Nature. 2017;543:252–6. 28219080 10.1038/nature21379 PMC5509051

[106] Camell C, Smith CW. Dietary oleic acid increases m2 macrophages in the mesenteric adipose tissue. PLoS One. 2013;8:e75147. 24098682 10.1371/journal.pone.0075147 PMC3787090

[107] Pardo V, Gonzalez-Rodriguez A, Guijas C, Balsinde J, Valverde AM. Opposite cross-talk by oleate and palmitate on insulin signaling in hepatocytes through macrophage activation. J Biol Chem. 2015;290:11663–77. 25792746 10.1074/jbc.M115.649483 PMC4416868

[108] Reilly NA, Sonnet F, Dekkers KF, Kwekkeboom JC, Sinke L, Hilt S, et al. Oleic acid triggers metabolic rewiring of T cells poising them for T helper 9 differentiation. iScience. 2024;27:109496. 38558932 10.1016/j.isci.2024.109496 PMC10981094

[109] von Hegedus JH, de Jong AJ, Hoekstra AT, Spronsen E, Zhu W, Cabukusta B, et al. Oleic acid enhances proliferation and calcium mobilization of CD3/CD28 activated CD4( + ) T cells through incorporation into membrane lipids. Eur J Immunol. 2024;54:e2350685. 38890809 10.1002/eji.202350685

[110] Manzo T, Prentice BM, Anderson KG, Raman A, Schalck A, Codreanu GS, et al. Accumulation of long-chain fatty acids in the tumor microenvironment drives dysfunction in intrapancreatic CD8 + T cells. J Exp Med. 2020;217. 10.1084/jem.20191920 PMC7398173 32491160

[111] Lu Z, Li Y, Jin J, Zhang X, Hannun YA, Huang Y. GPR40/FFA1 and neutral sphingomyelinase are involved in palmitate-boosted inflammatory response of microvascular endothelial cells to LPS. Atherosclerosis. 2015;240:163–73. 25795558 10.1016/j.atherosclerosis.2015.03.013 PMC4397186

[112] Panse M, Gerst F, Kaiser G, Teutsch CA, Dolker R, Wagner R, et al. Activation of extracellular signal-regulated protein kinases 1 and 2 (ERK1/2) by free fatty acid receptor 1 (FFAR1/GPR40) protects from palmitate-induced beta cell death, but plays no role in insulin secretion. Cell Physiol Biochem. 2015;35:1537–45. 25792236 10.1159/000373969

[113] Latruffe N, Vamecq J. Peroxisome proliferators and peroxisome proliferator activated receptors (PPARs) as regulators of lipid metabolism. Biochimie. 1997;79:81–94. 9209701 10.1016/s0300-9084(97)81496-4

[114] Chatelain F, Kohl C, Esser V, McGarry JD, Girard J, Pegorier JP. Cyclic AMP and fatty acids increase carnitine palmitoyltransferase I gene transcription in cultured fetal rat hepatocytes. Eur J Biochem. 1996;235:789–98. 8654430 10.1111/j.1432-1033.1996.00789.x

[115] Hostetler HA, McIntosh AL, Atshaves BP, Storey SM, Payne HR, Kier AB, et al. L-FABP directly interacts with PPARalpha in cultured primary hepatocytes. J Lipid Res. 2009;50:1663–75. 19289416 10.1194/jlr.M900058-JLR200 PMC2724054

[116] Nicholas DA, Zhang K, Hung C, Glasgow S, Aruni AW, Unternaehrer J, et al. Palmitic acid is a toll-like receptor 4 ligand that induces human dendritic cell secretion of IL-1beta. PLoS One. 2017;12:e0176793. 28463985 10.1371/journal.pone.0176793 PMC5413048

[117] Hwang DH, Kim JA, Lee JY. Mechanisms for the activation of Toll-like receptor 2/4 by saturated fatty acids and inhibition by docosahexaenoic acid. Eur J Pharm. 2016;785:24–35. 10.1016/j.ejphar.2016.04.024 PMC5815395 27085899

[118] Egnatchik RA, Leamy AK, Noguchi Y, Shiota M, Young JD. Palmitate-induced activation of mitochondrial metabolism promotes oxidative stress and apoptosis in H4IIEC3 rat hepatocytes. Metabolism. 2014;63:283–95. 24286856 10.1016/j.metabol.2013.10.009 PMC3946971

[119] Park J, Lee MY, Seo YS, Kang B, Lim SC, Kang KW. GPR40 agonist inhibits NLRP3 inflammasome activation via modulation of nuclear factor-kappaB and sarco/endoplasmic reticulum Ca(2 + )-ATPase. Life Sci. 2021;287:120127. 34774873 10.1016/j.lfs.2021.120127

[120] Souza PR, Walker ME, Goulding NJ, Dalli J, Perretti M, Norling LV. The GPR40 agonist GW9508 enhances neutrophil function to aid bacterial clearance during E. coli infections. Front Immunol. 2020;11:573019. 33133087 10.3389/fimmu.2020.573019 PMC7550532

[121] Recio C, Lucy D, Purvis GSD, Iveson P, Zeboudj L, Iqbal AJ, et al. Activation of the immune-metabolic receptor GPR84 enhances inflammation and phagocytosis in macrophages. Front Immunol. 2018;9:1419. 29973940 10.3389/fimmu.2018.01419 PMC6019444

[122] Liu, J, Liu, J, Qin, G, Li, J, Fu, Z, Li, J, et al. MDSCs-derived GPR84 induces CD8( + ) T-cell senescence via p53 activation to suppress the antitumor response. J Immunother Cancer. 2023;11:1–14. 10.1136/jitc-2023-007802 PMC10685939 38016719

[123] Oh DY, Lagakos WS. The role of G-protein-coupled receptors in mediating the effect of fatty acids on inflammation and insulin sensitivity. Curr Opin Clin Nutr Metab Care. 2011;14:322–7. 21587066 10.1097/MCO.0b013e3283479230

[124] Lu Z, Li Y, Syn WK, Li AJ, Ritter WS, Wank SA, et al. GPR40 deficiency is associated with hepatic FAT/CD36 upregulation, steatosis, inflammation, and cell injury in C57BL/6 mice. Am J Physiol Endocrinol Metab. 2021;320:E30–E42. 33103454 10.1152/ajpendo.00257.2020 PMC8436599

[125] Yan Y, Jiang W, Spinetti T, Tardivel A, Castillo R, Bourquin C, et al. Omega-3 fatty acids prevent inflammation and metabolic disorder through inhibition of NLRP3 inflammasome activation. Immunity. 2013;38:1154–63. 23809162 10.1016/j.immuni.2013.05.015

[126] Serhan CN. Novel omega - 3-derived local mediators in anti-inflammation and resolution. Pharm Ther. 2005;105:7–21. 10.1016/j.pharmthera.2004.09.002 15626453

[127] Schwab JM, Chiang N, Arita M, Serhan CN. Resolvin E1 and protectin D1 activate inflammation-resolution programmes. Nature. 2007;447:869–74. 17568749 10.1038/nature05877 PMC2757086

[128] Zhang S, Roth BL. Sensing unsaturated fatty acids: insights from GPR120 signaling. Cell Res. 2023;33:657–8. 37142674 10.1038/s41422-023-00814-2 PMC10474016

[129] Yu H, Yang W, Huang J, Miao X, Wang B, Ren X, et al. GPR120 induces regulatory dendritic cells by inhibiting HK2-dependent glycolysis to alleviate fulminant hepatic failure. Cell Death Dis. 2021;13:1. 34911928 10.1038/s41419-021-04394-0 PMC8674251

[130] Yang W, Liu H, Xu L, Yu T, Zhao X, Yao S, et al. GPR120 Inhibits Colitis Through Regulation of CD4( + ) T Cell Interleukin 10 Production. Gastroenterology. 2022;162:150–65. 34536451 10.1053/j.gastro.2021.09.018 PMC8678294

[131] Innes JK, Calder PC. Omega-6 fatty acids and inflammation. Prostaglandins Leukot Ess Fat Acids. 2018;132:41–48. 10.1016/j.plefa.2018.03.004 29610056

[132] Hansen HS, Rosenkilde MM, Holst JJ, Schwartz TW. GPR119 as a fat sensor. Trends Pharm Sci. 2012;33:374–81. 22560300 10.1016/j.tips.2012.03.014

[133] Overton HA, Fyfe MC, Reynet C. GPR119, a novel G protein-coupled receptor target for the treatment of type 2 diabetes and obesity. Br J Pharm. 2008;153:S76–81. 10.1038/sj.bjp.0707529 PMC2268073 18037923

[134] Mehdi SF, Pusapati S, Anwar MS, Lohana D, Kumar P, Nandula SA, et al. Glucagon-like peptide-1: a multi-faceted anti-inflammatory agent. Front Immunol. 2023;14:1148209. 37266425 10.3389/fimmu.2023.1148209 PMC10230051

[135] Ricchi M, Odoardi MR, Carulli L, Anzivino C, Ballestri S, Pinetti A, et al. Differential effect of oleic and palmitic acid on lipid accumulation and apoptosis in cultured hepatocytes. J Gastroenterol Hepatol. 2009;24:830–40. 19207680 10.1111/j.1440-1746.2008.05733.x

[136] Miller TA, LeBrasseur NK, Cote GM, Trucillo MP, Pimentel DR, Ido Y, et al. Oleate prevents palmitate-induced cytotoxic stress in cardiac myocytes. Biochem Biophys Res Commun. 2005;336:309–15. 16126172 10.1016/j.bbrc.2005.08.088

[137] Peng G, Li L, Liu Y, Pu J, Zhang S, Yu J, et al. Oleate blocks palmitate-induced abnormal lipid distribution, endoplasmic reticulum expansion and stress, and insulin resistance in skeletal muscle. Endocrinology. 2011;152:2206–18. 21505048 10.1210/en.2010-1369

[138] Oh DY, Talukdar S, Bae EJ, Imamura T, Morinaga H, Fan W, et al. GPR120 is an omega-3 fatty acid receptor mediating potent anti-inflammatory and insulin-sensitizing effects. Cell. 2010;142:687–98. 20813258 10.1016/j.cell.2010.07.041 PMC2956412

[139] Lu Z, Li Y, Li AJ, Syn WK, Wank SA, Lopes-Virella MF, et al. Loss of GPR40 in LDL receptor-deficient mice exacerbates high-fat diet-induced hyperlipidemia and nonalcoholic steatohepatitis. PLoS One. 2022;17:e0277251. 36331958 10.1371/journal.pone.0277251 PMC9635748

[140] Mao C, Xiao P, Tao XN, Qin J, He QT, Zhang C, et al. Unsaturated bond recognition leads to biased signal in a fatty acid receptor. Science. 2023;380:eadd6220. 36862765 10.1126/science.add6220

[141] Gerhart-Hines Z, Rodgers JT, Bare O, Lerin C, Kim SH, Mostoslavsky R, et al. Metabolic control of muscle mitochondrial function and fatty acid oxidation through SIRT1/PGC-1alpha. EMBO J. 2007;26:1913–23. 17347648 10.1038/sj.emboj.7601633 PMC1847661

[142] Tai KY, Chen CL, Fan SM, Kuan CH, Lin CK, Huang HW, et al. Adipocyte lipolysis activates epithelial stem cells for hair regeneration through fatty acid metabolic signaling. Cell Metab. 2025;37:2202–2219.e2208. 41130201 10.1016/j.cmet.2025.09.012

[143] Kim CH. Complex regulatory effects of gut microbial short-chain fatty acids on immune tolerance and autoimmunity. Cell Mol Immunol. 2023;20:341–50. 36854801 10.1038/s41423-023-00987-1 PMC10066346

[144] Richards JL, Yap YA, McLeod KH, Mackay CR, Marino E. Dietary metabolites and the gut microbiota: an alternative approach to control inflammatory and autoimmune diseases. Clin Transl Immunol. 2016;5:e82. 10.1038/cti.2016.29 PMC4910123 27350881

[145] Lukacs NW, Hogan SP. Food allergy: begin at the skin, end at the mast cell? Nat Rev Immunol. 2025. 10.1038/s41577-025-01185-y. 10.1038/s41577-025-01185-y PMC13044961 40571771

[146] Ogulur I, Mitamura Y, Yazici D, Pat Y, Ardicli S, Li M, et al. Type 2 immunity in allergic diseases. Cell Mol Immunol. 2025;22:211–42. 39962262 10.1038/s41423-025-01261-2 PMC11868591

[147] Huang MT, Chiu CJ, Tsai CY, Lee YR, Liu WL, Chuang HL, et al. Short-chain fatty acids ameliorate allergic airway inflammation via sequential induction of PMN-MDSCs and Treg cells. J Allergy Clin Immunol Glob. 2023;2:100163. 37781663 10.1016/j.jacig.2023.100163 PMC10509984

[148] Huang C, Du W, Ni Y, Lan G, Shi G. The effect of short-chain fatty acids on M2 macrophages polarization in vitro and in vivo. Clin Exp Immunol. 2022;207:53–64. 35020860 10.1093/cei/uxab028 PMC8802183

[149] Folkerts J, Redegeld F, Folkerts G, Blokhuis B, van den Berg MPM, de Bruijn MJW, et al. Butyrate inhibits human mast cell activation via epigenetic regulation of FcepsilonRI-mediated signaling. Allergy. 2020;75:1966–78. 32112426 10.1111/all.14254 PMC7703657

[150] Kim M, Friesen L, Park J, Kim HM, Kim CH. Microbial metabolites, short-chain fatty acids, restrain tissue bacterial load, chronic inflammation, and associated cancer in the colon of mice. Eur J Immunol. 2018;48:1235–47. 29644622 10.1002/eji.201747122 PMC6310065

[151] Sivaprakasam S, Gurav A, Paschall AV, Coe GL, Chaudhary K, Cai Y, et al. An essential role of Ffar2 (Gpr43) in dietary fibre-mediated promotion of healthy composition of gut microbiota and suppression of intestinal carcinogenesis. Oncogenesis. 2016;5:e238. 27348268 10.1038/oncsis.2016.38 PMC4945739

[152] Pan P, Skaer WC, Wang HT, Oshima K, Huang YW, Yu J, et al. Loss of free fatty acid receptor 2 enhances colonic adenoma development and reduces the chemopreventive effects of black raspberries in ApcMin/+ mice. Carcinogenesis. 2017;38:86–93. 27866157 10.1093/carcin/bgw122 PMC5219051

[153] Shiga H, Kajiura T, Shinozaki J, Takagi S, Kinouchi Y, Takahashi S, et al. Changes of faecal microbiota in patients with Crohn’s disease treated with an elemental diet and total parenteral nutrition. Dig Liver Dis. 2012;44:736–42. 22622202 10.1016/j.dld.2012.04.014

[154] Joossens M, Huys G, Cnockaert M, De Preter V, Verbeke K, Rutgeerts P, et al. Dysbiosis of the faecal microbiota in patients with Crohn’s disease and their unaffected relatives. Gut. 2011;60:631–7. 21209126 10.1136/gut.2010.223263

[155] Jackman JA, Boyd RD, Elrod CC. Medium-chain fatty acids and monoglycerides as feed additives for pig production: towards gut health improvement and feed pathogen mitigation. J Anim Sci Biotechnol. 2020;11:44. 32337029 10.1186/s40104-020-00446-1 PMC7178611

[156] Hoshimoto A, Suzuki Y, Katsuno T, Nakajima H, Saito Y. Caprylic acid and medium-chain triglycerides inhibit IL-8 gene transcription in Caco-2 cells: comparison with the potent histone deacetylase inhibitor trichostatin A. Br J Pharm. 2002;136:280–6. 10.1038/sj.bjp.0704719 PMC1573354 12010777

[157] Haghikia A, Jorg S, Duscha A, Berg J, Manzel A, Waschbisch A, et al. Dietary fatty acids directly impact central nervous system autoimmunity via the small intestine. Immunity. 2015;43:817–29. 26488817 10.1016/j.immuni.2015.09.007

[158] Hammer A, Schliep A, Jorg S, Haghikia A, Gold R, Kleinewietfeld M, et al. Impact of combined sodium chloride and saturated long-chain fatty acid challenge on the differentiation of T helper cells in neuroinflammation. J Neuroinflammat. 2017;14:184. 10.1186/s12974-017-0954-y PMC5596846 28899400

[159] Park J, Wang Q, Wu Q, Mao-Draayer Y, Kim CH. Bidirectional regulatory potentials of short-chain fatty acids and their G-protein-coupled receptors in autoimmune neuroinflammation. Sci Rep. 2019;9:8837. 31222050 10.1038/s41598-019-45311-y PMC6586800

[160] Saresella M, Marventano I, Barone M, La Rosa F, Piancone F, Mendozzi L, et al. Alterations in circulating fatty acid are associated with gut microbiota dysbiosis and inflammation in multiple sclerosis. Front Immunol. 2020;11:1390. 32733460 10.3389/fimmu.2020.01390 PMC7358580

[161] De Preter V, Machiels K, Joossens M, Arijs I, Matthys C, Vermeire S, et al. Faecal metabolite profiling identifies medium-chain fatty acids as discriminating compounds in IBD. Gut. 2015;64:447–58. 24811995 10.1136/gutjnl-2013-306423

[162] Xu C, Liu Q, Xu M, Ayalew H, Iqbal W, Lin J, et al. Dietary medium-chain fatty acids mitigate Escherichia coli O78 infection in broilers by enhancing intestinal immune barrier and modulating microbiota. Poult Sci. 2025;104:105775. 40961776 10.1016/j.psj.2025.105775 PMC12475841

[163] Laroui H, Ingersoll SA, Liu HC, Baker MT, Ayyadurai S, Charania MA, et al. Dextran sodium sulfate (DSS) induces colitis in mice by forming nano-lipocomplexes with medium-chain-length fatty acids in the colon. PLoS One. 2012;7:e32084. 22427817 10.1371/journal.pone.0032084 PMC3302894

[164] Toms TE, Panoulas VF, Kitas GD. Dyslipidaemia in rheumatological autoimmune diseases. Open Cardiovasc Med J. 2011;5:64–75. 21660202 10.2174/1874192401105010064 PMC3109701

[165] Kim SY, Yu M, Morin EE, Kang J, Kaplan MJ, Schwendeman A. High-density lipoprotein in lupus: disease biomarkers and potential therapeutic strategy. Arthritis Rheumatol. 2020;72:20–30. 31350818 10.1002/art.41059 PMC6935404

[166] Wu T, Xie C, Han J, Ye Y, Weiel J, Li Q, et al. Metabolic disturbances associated with systemic lupus erythematosus. PLoS One. 2012;7:e37210. 22723834 10.1371/journal.pone.0037210 PMC3378560

[167] Zhao L, Kwon MJ, Huang S, Lee JY, Fukase K, Inohara N, et al. Differential modulation of Nods signaling pathways by fatty acids in human colonic epithelial HCT116 cells. J Biol Chem. 2007;282:11618–28. 17303577 10.1074/jbc.M608644200

[168] Reddy KVK, Naidu KA. Oleic acid, hydroxytyrosol and n-3 fatty acids collectively modulate colitis through reduction of oxidative stress and IL-8 synthesis; in vitro and in vivo studies. Int Immunopharmacol. 2016;35:29–42. 27016717 10.1016/j.intimp.2016.03.019

[169] Ji Z, Wu S, Xu Y, Qi J, Su X, Shen L. Obesity promotes EAE through IL-6 and CCL-2-mediated T cells infiltration. Front Immunol. 2019;10:1881. 31507583 10.3389/fimmu.2019.01881 PMC6718738

[170] Timmermans S, Bogie JF, Vanmierlo T, Lutjohann D, Stinissen P, Hellings N, et al. High fat diet exacerbates neuroinflammation in an animal model of multiple sclerosis by activation of the Renin Angiotensin system. J Neuroimmune Pharm. 2014;9:209–17. 10.1007/s11481-013-9502-4 24068577

[171] Miles EA, Calder PC. Influence of marine n-3 polyunsaturated fatty acids on immune function and a systematic review of their effects on clinical outcomes in rheumatoid arthritis. Br J Nutr. 2012;107:S171–184. 22591891 10.1017/S0007114512001560

[172] Hawthorne AB, Daneshmend TK, Hawkey CJ, Belluzzi A, Everitt SJ, Holmes GK, et al. Treatment of ulcerative colitis with fish oil supplementation: a prospective 12 month randomised controlled trial. Gut. 1992;33:922–8. 1353742 10.1136/gut.33.7.922 PMC1379405

[173] Shoda R, Matsueda K, Yamato S, Umeda N. Epidemiologic analysis of Crohn disease in Japan: increased dietary intake of n-6 polyunsaturated fatty acids and animal protein relates to the increased incidence of Crohn disease in Japan. Am J Clin Nutr. 1996;63:741–5. 8615358 10.1093/ajcn/63.5.741

[174] Brigham EP, Woo H, McCormack M, Rice J, Koehler K, Vulcain T, et al. Omega-3 and Omega-6 intake modifies asthma severity and response to indoor air pollution in children. Am J Respir Crit Care Med. 2019;199:1478–86. 30922077 10.1164/rccm.201808-1474OC PMC6580674

[175] Barebring L, Nwaru BI, Lamberg-Allardt C, Thorisdottir B, Ramel A, Soderlund F, et al. Supplementation with long chain n-3 fatty acids during pregnancy, lactation, or infancy in relation to risk of asthma and atopic disease during childhood: a systematic review and meta-analysis of randomized controlled clinical trials. Food Nutr Res. 2022;66:1–13. 10.29219/fnr.v66.8842 PMC9602204 36340915

[176] van den Elsen LW, Bol-Schoenmakers M, van Esch BC, Hofman GA, van de Heijning BJ, Pieters RH, et al. DHA-rich tuna oil effectively suppresses allergic symptoms in mice allergic to whey or peanut. J Nutr. 2014;144:1970–6. 25342698 10.3945/jn.114.198515

[177] Cawood AL, Ding R, Napper FL, Young RH, Williams JA, Ward MJ, et al. Eicosapentaenoic acid (EPA) from highly concentrated n-3 fatty acid ethyl esters is incorporated into advanced atherosclerotic plaques and higher plaque EPA is associated with decreased plaque inflammation and increased stability. Atherosclerosis. 2010;212:252–9. 20542512 10.1016/j.atherosclerosis.2010.05.022

[178] Vogel FCE, Chaves-Filho AB, Schulze A. Lipids as mediators of cancer progression and metastasis. Nat Cancer. 2024;5:16–29. 38273023 10.1038/s43018-023-00702-z

[179] Jin R, Hao J, Yi Y, Yin D, Hua Y, Li X, et al. Dietary fats high in linoleic acids impair antitumor T-cell responses by inducing E-FABP-mediated mitochondrial dysfunction. Cancer Res. 2021;81:5296–310. 34400394 10.1158/0008-5472.CAN-21-0757 PMC8530923

[180] Nava Lauson CB, Tiberti S, Corsetto PA, Conte F, Tyagi P, Machwirth M, et al. Linoleic acid potentiates CD8( + ) T cell metabolic fitness and antitumor immunity. Cell Metab. 2023;35:633–50.e639. 36898381 10.1016/j.cmet.2023.02.013

[181] Cani PD, Neyrinck AM, Maton N, Delzenne NM. Oligofructose promotes satiety in rats fed a high-fat diet: involvement of glucagon-like Peptide-1. Obes Res. 2005;13:1000–7. 15976142 10.1038/oby.2005.117

[182] Tolhurst G, Heffron H, Lam YS, Parker HE, Habib AM, Diakogiannaki E, et al. Short-chain fatty acids stimulate glucagon-like peptide-1 secretion via the G-protein-coupled receptor FFAR2. Diabetes. 2012;61:364–71. 22190648 10.2337/db11-1019 PMC3266401

[183] Cherbut C, Ferrier L, Roze C, Anini Y, Blottiere H, Lecannu G, et al. Short-chain fatty acids modify colonic motility through nerves and polypeptide YY release in the rat. Am J Physiol. 1998;275:G1415–1422. 9843779 10.1152/ajpgi.1998.275.6.G1415

[184] Frost G, Sleeth ML, Sahuri-Arisoylu M, Lizarbe B, Cerdan S, Brody L, et al. The short-chain fatty acid acetate reduces appetite via a central homeostatic mechanism. Nat Commun. 2014;5:3611. 24781306 10.1038/ncomms4611 PMC4015327

[185] Kimura I, Inoue D, Maeda T, Hara T, Ichimura A, Miyauchi S, et al. Short-chain fatty acids and ketones directly regulate sympathetic nervous system via G protein-coupled receptor 41 (GPR41). Proc Natl Acad Sci USA. 2011;108:8030–5. 21518883 10.1073/pnas.1016088108 PMC3093469

[186] Fushimi T, Tayama K, Fukaya M, Kitakoshi K, Nakai N, Tsukamoto Y, et al. Acetic acid feeding enhances glycogen repletion in liver and skeletal muscle of rats. J Nutr. 2001;131:1973–7. 11435516 10.1093/jn/131.7.1973

[187] Zhao L, Zhang F, Ding X, Wu G, Lam YY, Wang X, et al. Gut bacteria selectively promoted by dietary fibers alleviate type 2 diabetes. Science. 2018;359:1151–6. 29590046 10.1126/science.aao5774

[188] Qin J, Li Y, Cai Z, Li S, Zhu J, Zhang F, et al. A metagenome-wide association study of gut microbiota in type 2 diabetes. Nature. 2012;490:55–60. 23023125 10.1038/nature11450

[189] Karlsson FH, Tremaroli V, Nookaew I, Bergstrom G, Behre CJ, Fagerberg B, et al. Gut metagenome in European women with normal, impaired and diabetic glucose control. Nature. 2013;498:99–103. 23719380 10.1038/nature12198

[190] Chandalia M, Garg A, Lutjohann D, von Bergmann K, Grundy SM, Brinkley LJ. Beneficial effects of high dietary fiber intake in patients with type 2 diabetes mellitus. N Engl J Med. 2000;342:1392–8. 10805824 10.1056/NEJM200005113421903

[191] Silva FM, Kramer CK, Crispim D, Azevedo MJ. A high-glycemic index, low-fiber breakfast affects the postprandial plasma glucose, insulin, and ghrelin responses of patients with type 2 diabetes in a randomized clinical trial. J Nutr. 2015;145:736–41. 25833777 10.3945/jn.114.195339

[192] Bell KJ, Saad S, Tillett BJ, McGuire HM, Bordbar S, Yap YA, et al. Metabolite-based dietary supplementation in human type 1 diabetes is associated with microbiota and immune modulation. Microbiome. 2022;10:9. 35045871 10.1186/s40168-021-01193-9 PMC8772108

[193] Tillett BJ, Dwiyanto J, Secombe KR, George T, Zhang V, Anderson D, et al. SCFA biotherapy delays diabetes in humanized gnotobiotic mice by remodeling mucosal homeostasis and metabolome. Nat Commun. 2025;16:2893. 40133336 10.1038/s41467-025-58319-y PMC11937418

[194] Munte E, Viebahn G, Khurana A, Fujiki J, Nakamura T, Lang S et al. Faecalibacterium prausnitzii Is Associated with Disease Severity in MASLD but Its Supplementation Does Not Improve Diet-Induced Steatohepatitis in Mice. Microorganisms. 2015;13. 10.3390/microorganisms13030675 PMC11944644 40142567

[195] Brooks L, Viardot A, Tsakmaki A, Stolarczyk E, Howard JK, Cani PD, et al. Fermentable carbohydrate stimulates FFAR2-dependent colonic PYY cell expansion to increase satiety. Mol Metab. 2017;6:48–60. 28123937 10.1016/j.molmet.2016.10.011 PMC5220466

[196] Freeland KR, Wilson C, Wolever TM. Adaptation of colonic fermentation and glucagon-like peptide-1 secretion with increased wheat fibre intake for 1 year in hyperinsulinaemic human subjects. Br J Nutr. 2010;103:82–90. 19664300 10.1017/S0007114509991462

[197] Lin HV, Frassetto A, Kowalik EJ Jr, Nawrocki AR, Lu MM, Kosinski JR, et al. Butyrate and propionate protect against diet-induced obesity and regulate gut hormones via free fatty acid receptor 3-independent mechanisms. PLoS One. 2012;7:e35240. 22506074 10.1371/journal.pone.0035240 PMC3323649

[198] Psichas A, Sleeth ML, Murphy KG, Brooks L, Bewick GA, Hanyaloglu AC, et al. The short chain fatty acid propionate stimulates GLP-1 and PYY secretion via free fatty acid receptor 2 in rodents. Int J Obes (Lond). 2015;39:424–9. 25109781 10.1038/ijo.2014.153 PMC4356745

[199] van der Beek CM, Canfora EE, Lenaerts K, Troost FJ, Damink S, Holst JJ, et al. Distal, not proximal, colonic acetate infusions promote fat oxidation and improve metabolic markers in overweight/obese men. Clin Sci (Lond). 2016;130:2073–82. 27439969 10.1042/CS20160263

[200] Kondo T, Kishi M, Fushimi T, Kaga T. Acetic acid upregulates the expression of genes for fatty acid oxidation enzymes in liver to suppress body fat accumulation. J Agric Food Chem. 2009;57:5982–6. 19469536 10.1021/jf900470c

[201] Gao Z, Yin J, Zhang J, Ward RE, Martin RJ, Lefevre M, et al. Butyrate improves insulin sensitivity and increases energy expenditure in mice. Diabetes. 2009;58:1509–17. 19366864 10.2337/db08-1637 PMC2699871

[202] Park JS, Lee EJ, Lee JC, Kim WK, Kim HS. Anti-inflammatory effects of short chain fatty acids in IFN-gamma-stimulated RAW 264.7 murine macrophage cells: involvement of NF-kappaB and ERK signaling pathways. Int Immunopharmacol. 2007;7:70–77. 17161819 10.1016/j.intimp.2006.08.015

[203] Montgomery MK, Osborne B, Brown SH, Small L, Mitchell TW, Cooney GJ, et al. Contrasting metabolic effects of medium- versus long-chain fatty acids in skeletal muscle. J Lipid Res. 2013;54:3322–33. 24078708 10.1194/jlr.M040451 PMC3826680

[204] Cavaleri F, Bashar E. Potential synergies of beta-hydroxybutyrate and butyrate on the modulation of metabolism, inflammation, cognition, and general health. J Nutr Metab. 2018;2018:7195760. 29805804 10.1155/2018/7195760 PMC5902005

[205] Peiris M, Aktar R, Reed D, Cibert-Goton V, Zdanaviciene A, Halder W, et al. Decoy bypass for appetite suppression in obese adults: role of synergistic nutrient sensing receptors GPR84 and FFAR4 on colonic endocrine cells. Gut. 2022;71:928–37. 34083384 10.1136/gutjnl-2020-323219 PMC8995825

[206] Cao Y, Araki M, Nakagawa Y, Deisen L, Lundsgaard A, Kanta JM, et al. Dietary medium-chain fatty acids reduce hepatic fat accumulation via activation of a CREBH-FGF21 axis. Mol Metab. 2024;87:101991. 39019116 10.1016/j.molmet.2024.101991 PMC11327439

[207] Manzel A, Muller DN, Hafler DA, Erdman SE, Linker RA, Kleinewietfeld M. Role of “Western diet“ in inflammatory autoimmune diseases. Curr Allergy Asthma Rep. 2014;14:404. 24338487 10.1007/s11882-013-0404-6 PMC4034518

[208] Shin S. Regulation of adipose tissue biology by long-chain fatty acids: metabolic effects and molecular mechanisms. J Obes Metab Syndr. 2022;31:147–60. 35691686 10.7570/jomes22014 PMC9284576

[209] Karsten S, Schafer G, Schauder P. Cytokine production and DNA synthesis by human peripheral lymphocytes in response to palmitic, stearic, oleic, and linoleic acid. J Cell Physiol. 1994;161:15–22. 7929601 10.1002/jcp.1041610103

[210] Stentz FB, Kitabchi AE. Palmitic acid-induced activation of human T-lymphocytes and aortic endothelial cells with production of insulin receptors, reactive oxygen species, cytokines, and lipid peroxidation. Biochem Biophys Res Commun. 2006;346:721–6. 16782068 10.1016/j.bbrc.2006.05.159

[211] Haghikia A, Jorg S, Duscha A, Berg J, Manzel A, Waschbisch A, et al. Dietary fatty acids directly impact central nervous system autoimmunity via the small intestine. Immunity. 2016;44:951–3. 27096322 10.1016/j.immuni.2016.04.006

[212] Gioia C, Lucchino B, Tarsitano MG, Iannuccelli C, Di Franco, M. Dietary habits and nutrition in rheumatoid arthritis: can diet influence disease development and clinical manifestations? Nutrients. 2020;12:1–25. 10.3390/nu12051456 PMC7284442 32443535

[213] Czauderna A, Kulkarni G, Bianchi N, Cheng L, Sim M, Realini NR, et al. Long-chain unsaturated fatty acids released during immune responses stimulate host-microbe trans-kingdom communication. Cell Host Microbe. 2025;33:1667–1685.e1614. 40972570 10.1016/j.chom.2025.08.011

[214] de Wit N, Derrien M, Bosch-Vermeulen H, Oosterink E, Keshtkar S, Duval C, et al. Saturated fat stimulates obesity and hepatic steatosis and affects gut microbiota composition by an enhanced overflow of dietary fat to the distal intestine. Am J Physiol Gastrointest Liver Physiol. 2012;303:G589–599. 22700822 10.1152/ajpgi.00488.2011

[215] Mujico JR, Baccan GC, Gheorghe A, Diaz LE, Marcos A. Changes in gut microbiota due to supplemented fatty acids in diet-induced obese mice. Br J Nutr. 2013;110:711–20. 23302605 10.1017/S0007114512005612

[216] Schoeler M, Ellero-Simatos S, Birkner T, Mayneris-Perxachs J, Olsson L, Brolin H, et al. The interplay between dietary fatty acids and gut microbiota influences host metabolism and hepatic steatosis. Nat Commun. 2023;14:5329. 37658064 10.1038/s41467-023-41074-3 PMC10474162

[217] Alcock J, Lin HC. Fatty acids from diet and microbiota regulate energy metabolism. F1000Res. 2015;4:738. 27006755 10.12688/f1000research.6078.1 PMC4797936

[218] Machate DJ, Figueiredo PS, Marcelino G, Guimaraes RCA, Hiane PA, Bogo D et al. Fatty acid diets: regulation of gut microbiota composition and obesity and its related metabolic dysbiosis. Int J Mol Sci. 2020;21. 10.3390/ijms21114093 PMC7312778 32521778

[219] Tangwatcharin P, Khopaibool P. Activity of virgin coconut oil, lauric acid or monolaurin in combination with lactic acid against Staphylococcus aureus. Southeast Asian J Trop Med Public Health. 2012;43:969–85. 23077821

[220] Hannun YA, Obeid LM. Principles of bioactive lipid signalling: lessons from sphingolipids. Nat Rev Mol Cell Biol. 2008;9:139–50. 18216770 10.1038/nrm2329

[221] Passani MB, Provensi G, Piomelli D. Editorial: the paracannabinoid system: endocannabinoid-like lipids and their functions. Front Endocrinol (Lausanne). 2023;14:1263924. 38027133 10.3389/fendo.2023.1263924 PMC10646612

[222] Dennis EA, Norris PC. Eicosanoid storm in infection and inflammation. Nat Rev Immunol. 2015;15:511–23. 26139350 10.1038/nri3859 PMC4606863

[223] Calder PC. Omega-3 fatty acids and inflammatory processes: from molecules to man. Biochem Soc Trans. 2017;45:1105–15. 28900017 10.1042/BST20160474

[224] Serhan CN. Pro-resolving lipid mediators are leads for resolution physiology. Nature. 2014;510:92–101. 24899309 10.1038/nature13479 PMC4263681

[225] Fan F, Roman RJ. GPR75 identified as the first 20-HETE receptor: a chemokine receptor adopted by a new family. Circ Res. 2017;120:1696–8. 28546348 10.1161/CIRCRESAHA.117.311022 PMC5766006

